# The Role of Cold-Inducible RNA-Binding Protein (CIRP) in Neurological Disorders

**DOI:** 10.3390/brainsci16020205

**Published:** 2026-02-09

**Authors:** Xueqi Lai, Peng Zhong

**Affiliations:** Department of Cardiology, Renmin Hospital of Wuhan University, Wuhan 430060, China; 2022305231078@whu.edu.cn

**Keywords:** cold-inducible RNA-binding protein (CIRP), hypothermic brain protection, stroke, traumatic brain injury, Alzheimer’s disease, neuroinflammation, damage-associated molecular patterns (DAMPs)

## Abstract

Cold-inducible RNA-binding protein (CIRP) is a critical molecule in the central nervous system (CNS) with functions that depend on its subcellular localization, exhibiting biphasic regulatory roles in both physiological and pathological processes. Under physiological conditions, intracellular cold-inducible RNA-binding protein (iCIRP) contributes to the maintenance of circadian rhythms by regulating the stability of core clock gene mRNAs and exerts neuroprotective effects during mild hypothermia by preserving the blood–brain barrier and inhibiting apoptosis. Pathologically, extracellular cold-inducible RNA-binding protein (eCIRP) functions as a damage-associated molecular pattern (DAMP) that drives neuroinflammation and brain injury. In ischemic stroke (IS), eCIRP promotes neutrophil extracellular trap (NET) formation and increases microglial activity via the Toll-like receptor 4 (TLR4) pathway. In cerebral ischemia–reperfusion (I/R) injury, eCIRP activates oxidative stress and the NOD-like receptor thermal protein domain associated protein 3 (NLRP3) inflammasome through the TLR4 axis, exacerbating mitochondrial damage. In intracerebral hemorrhage (ICH), eCIRP further amplifies inflammation via the interleukin-6 receptor (IL-6R)/signal transducer and activator of transcription 3 (STAT3) signaling pathway. In traumatic brain injury (TBI), eCIRP activates the endoplasmic reticulum stress pathway, intensifying apoptosis. In Alzheimer’s disease (AD), eCIRP regulates tau phosphorylation and β-amyloid (Aβ) metabolism and may mediate the link between alcohol exposure and AD pathology. Preclinical studies indicate that serum eCIRP levels correlate with IS and ICH severity, highlighting its potential as a biomarker. This systematic review elucidates the mechanisms of CIRP in CNS diseases, providing insights for understanding and preventing conditions such as IS, cerebral I/R injury, ICH, TBI, and AD.

## 1. Introduction

Cold-inducible RNA-binding protein (CIRP), also known as CIRBP or heterogeneous nuclear ribonucleoprotein A18 (hnRNP A18), was first identified and cloned by Nishiyama et al. in 1997 as a key member of the cold shock protein family, with a molecular weight of ~18 kDa [1,2]. This protein comprises 172 amino acids and contains a highly conserved N-terminal RNA recognition motif (RRM) as well as a C-terminal glycine-rich region [1,2]. CIRP was initially identified for its upregulation of expression in response to low-temperature stimulation [2]. Subsequent studies have demonstrated that the regulation of its expression is not only influenced by low temperatures but can also be induced by a variety of stressors, including hypoxia, ultraviolet radiation, energy deprivation, and heat shock [3].

As a stress-responsive protein, CIRP exhibits significantly different biological functions in both intracellular and extracellular environments. Under steady-state conditions, CIRP resides within the cell nucleus as intracellular cold-inducible RNA-binding protein (iCIRP), involved in essential biological processes such as maintaining cellular homeostasis, regulating circadian rhythms, protecting telomere stability, and influencing tumorigenesis by modulating mRNA stability [4,5]. By selectively binding to the 3′ untranslated region (3′ UTR) of target mRNAs, CIRP precisely modulates their stability and translation efficiency, thereby influencing cell cycle progression and metabolic activities [4]. Notably, under pathological conditions such as hypoxia, ischemia, or oxidative stress, CIRP not only exhibits significantly elevated levels but also undergoes nucleoplasmic translocation, becoming enriched in stress granules and ultimately entering the extracellular space through active secretion or passive release [3,6]. Extracellular cold-inducible RNA-binding protein (eCIRP) is a novel damage-associated molecular pattern (DAMP) that can trigger the nuclear factor kappa-B (NF-κB) signaling cascade by binding to and activating the Toll-like receptor 4 (TLR4)/Myeloid differentiation protein-2 (MD2) complex and the Triggering receptor expressed on myeloid cells-1 (TREM-1) receptor pathway, inducing the release of pro-inflammatory factors [7,8,9,10]. Studies have demonstrated that eCIRP plays a crucial role in the inflammatory pathology of disease models such as sepsis, ischemia–reperfusion injury, acute respiratory distress syndrome, and malignancy, suggesting its significance as a key inflammatory mediator in these conditions [9,11,12,13].

Brain tissue, as the fundamental structure of the central nervous system, regulates advanced cognitive and physiological functions through its intricate neural network. Injury to brain tissue can result in severe consequences, including cognitive dysfunction, motor disorders, and affective disorders. Recent studies have indicated that the protective effect of hypothermia therapy on brain injury may, in part, depend on the regulatory function of iCIRP. In fact, CIRP exhibits a distinct, spatially specific dual role in brain injury-related diseases: intracellular CIRP exerts protective effects on neuronal cells by maintaining the blood–brain barrier and resisting neuronal apoptosis [14,15,16,17,18,19,20], while extracellularly released CIRP acts as a DAMP, exacerbating neuroinflammatory responses and promoting neuronal apoptosis through pathways such as TLR4 signaling axis [21,22,23,24,25,26,27]. Currently, the pathological effects of eCIRP have been validated in a variety of brain disease models, including ischemic stroke (IS), intracerebral hemorrhage (ICH), traumatic brain injury (TBI), and neurodegenerative diseases. This review systematically analyzes the dual regulatory roles of CIRP in the pathological processes of brain injury and explores its mechanisms of action and translational medical value.

## 2. The Role of CIRP in the Regulation of Circadian Rhythms

CIRP, a highly conserved RNA-binding molecule throughout evolution, is widely expressed in various tissues, including the brain, heart, kidney, and immune system, in both humans and rodents [3]. Its role in regulating circadian rhythms has garnered broad research consensus. At the species level, CIRP exhibits highly conserved circadian expression patterns: both amphibians (African clawed frog [28]) and mammals (mouse [29]) display diurnal fluctuations in the suprachiasmatic nucleus (SCN)—the mammalian circadian center—and cortical regions, suggesting that CIRP functions as a fundamental regulatory mediator in the evolutionary development of biological clock systems.

CIRP expression demonstrates both tissue specificity and correlation with physiological states: rhythmic fluctuations are pronounced in mouse brain tissue, whereas expression remains stable in peripheral tissues such as the testes and liver [29]. Additionally, CIRP expression in the brain is closely associated with sleep homeostasis, exhibiting elevated levels during resting states and downregulation following sleep deprivation [30]. This pattern indicates its role in the coordinated regulation of circadian rhythms and sleep homeostasis. In vitro experiments further confirm that CIRP is a key regulator in maintaining high-amplitude rhythms in neurons [31], providing essential evidence for mechanistic studies.

### 2.1. Molecular Mechanisms of CIRP in Regulating Circadian Rhythms

CIRP maintains circadian homeostasis through “signal response–post-transcriptional regulation”. The light-responsive elements of retinal ganglion cells synchronously activate the expression of circadian-related genes and CIRP [32]. This coordinated molecular activation ensures efficient transmission of external photoperiod signals to the intrinsic rhythmic system. As an exogenous synchronizing signal, light is detected by intrinsically photosensitive retinal ganglion cells (ipRGCs) that express the photopigment melanopsin and is transmitted to the SCN via the retinal-hypothalamic tract (RHT) [33,34,35]. This mechanism has been validated in rat and mouse models, including genetically engineered mice, using immunostaining, neural tracing, and electrophysiological recording techniques [33,34,35]. Upon receiving light signals, the SCN drives rhythmic CIRP expression by regulating the 24 h fluctuations of core body temperature [31,36]. Prolonged darkness (absence of light signals) completely abolishes CIRP rhythms in adult mouse brains [29], confirming light’s regulatory role in CIRP rhythms. Studies on National Institutes of Health 3-day transfer, inoculum 3 × 10^5^ cells (NIH3T3) fibroblasts show that CIRP mRNA and protein exhibit 24 h periodic expression in cells synchronized by physiological temperature rhythms (38 °C to 34 °C), whereas CIRP rhythms completely disappear in cells maintained at a constant 36.5 °C after serum shock synchronization [31]. These findings demonstrate that CIRP rhythms are not directly regulated by circadian rhythms but depend on sustained temperature cycles, with body temperature signals serving as the core downstream driver.

As an RNA-binding protein, CIRP precisely regulates and amplifies effects within rhythmic networks through post-transcriptional control. CIRP exerts dual regulatory functions by directly binding to the 3′ untranslated regions (3′ UTRs) of circadian gene transcripts (e.g., circadian locomotor output cycles kaput (CLOCK), protein kinase R-like endoplasmic reticulum kinase (Per), and D-box binding PAR bZIP transcription factor (Dbp) mRNAs), thereby enhancing mRNA stability and promoting translation [31]. This mechanism has been validated through CLIP-seq and functional experiments conducted in NIH3T3 fibroblasts [31]. Gene silencing experiments conducted in NIH3T3 fibroblasts have demonstrated that CIRP deficiency significantly downregulates key circadian regulators, including CLOCK, silent information regulator 1 (SIRT1), and retinoic acid receptor-related orphan receptor-α (RORα) [31]. This indicates that CIRP enhances the amplitude of circadian oscillations by increasing the cytoplasmic accumulation of CLOCK mRNA and other potential circadian timing system regulators, such as RORα, nuclear receptor corepressor 1 (NCOR1), SIRT1, and period circadian regulator 3 (PER3). These findings establish CIRP as an essential factor for maintaining normal circadian rhythms, with its dysfunction potentially causing direct disruption of the rhythmic regulatory network.

### 2.2. Circadian Rhythm Disruption (CRD) as a Convergent Risk Factor for Neurodegenerative Diseases

Recent studies confirm that circadian rhythm disruption (CRD) is a key pathological mechanism underlying the progression of neurodegenerative diseases such as Alzheimer’s disease (AD), Parkinson’s disease (PD), Huntington’s disease (HD), and other neurodegenerative disorders [37,38]. The characteristics of CRD vary across these diseases. In AD patients, CRD exhibits stage-dependent patterns, with moderate-to-severe cases showing rhythm fragmentation, reduced amplitude, and sundowning syndrome, typically manifesting as irregular sleep–wake rhythm disorder (ISWRD) [39,40,41,42,43]. In PD patients, reduced rhythm amplitude is a central feature, with up to 80% experiencing sleep–wake disturbances such as excessive daytime sleepiness [44,45,46,47,48,49,50]. CRD is also prevalent in patients with multiple system atrophy (MSA), dementia with Lewy bodies (DLB), and frontotemporal dementia (FTD). For instance, CRD in MSA arises from autonomic network degeneration [51], while DLB patients exhibit reduced nocturnal core body temperature amplitude and high rates of rapid eye movement sleep behavior disorder (RBD) [52,53]. Conversely, FTD patients demonstrate phase-advanced rhythms [54].

Therefore, as a key regulator of circadian rhythms, abnormal expression or dysfunction of CIRP may promote the progression of neurodegenerative diseases by disrupting the biological clock. Research indicates that disruption of normal circadian rhythms can contribute to neurodegenerative changes in the brain through multiple mechanisms, including oxidative stress, inflammation, glial cell activation, impaired autophagy, and compromised lymphatic system function [55,56]. Specifically, circadian disruption in Alzheimer’s disease (AD) patients may increase β-amyloid (Aβ) accumulation and reduce hippocampal memory formation by abnormally regulating autophagy [57,58]. It may also promote Aβ and tau aggregation by activating astrocytes, disrupting the rhythmic expression of aquaporin 4 (AQP4), impairing lymphatic flow, and reducing the expression of proteins involved in Aβ uptake and degradation [59]. Concurrently, this process triggers neuroinflammation, exacerbating AD pathology. Based on these findings, modulating CIRP expression or activity through pharmacological or gene therapy approaches may represent a novel strategy for restoring circadian rhythms and ameliorating neurodegenerative disease pathology.

## 3. The Role of CIRP in Hypothermic Brain Protection

Although cold-induced stress responses can exacerbate certain pathological processes, controlled hypothermia shows significant therapeutic potential in specific clinical contexts, particularly in tissue preservation and central nervous system protection. Hypothermia-mediated neuroprotection primarily involves three mechanisms: reducing tissue metabolic rate, alleviating cerebral edema formation, and stabilizing the blood–brain barrier structure [60]. In recent years, iCIRP has been identified as a key regulatory factor in hypothermia-mediated neuroprotection.

### 3.1. CIRP-Mediated Neuroprotective Mechanisms at Mild Hypothermic Temperatures

**Figure 1:**
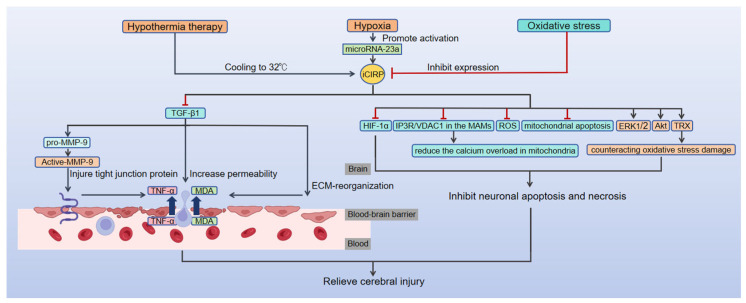
The intracellular protective role of CIRP in hypothermic brain protection. Intracellular CIRP expression is regulated by hypothermia, hypoxia, and oxidative stress. During CPB at 32 °C, hypothermia upregulates CIRP in brain tissue. CIRP downregulates TGF-β1 expression and maintains the integrity of the blood–brain barrier by inhibiting TGF-β1-mediated activation of MMP-9, thereby reducing pathologic deposition of extracellular matrix, which in turn decreases inflammatory cell infiltration and the release of TNF-α and MDA, ultimately attenuating neuronal injury. Furthermore, CIRP can mitigate brain injury by inhibiting neuronal apoptosis and counteracting oxidative stress damage through multiple pathways, thereby mitigating brain injury. TGF-β1: Transforming growth factor-β1; MMP-9: matrix metalloproteinase-9; TNF-α: tumor necrosis factor-α; MDA: malondialdehyde; HIF-1α: hypoxia-inducible factor-1α; MAM: mitochondria-associated endoplasmic reticulum membrane; IP3R: inositol 1,4,5-trisphosphate receptor; VDAC1: voltage-dependent anion channel 1; ROS: reactive oxygen species; ERK1/2: extracellular signal-regulated kinase; Akt: protein kinase B; TRX: thioredoxin.

Under mild hypothermic conditions, iCIRP exerts neuroprotective effects through multiple mechanisms, including inhibition of the transforming growth factor-β1 (TGF-β1)/Matrix metalloproteinase-9 (MMP-9) axis to preserve the blood–brain barrier [14], antagonism of neuronal apoptosis [15,16,17,18,19,20,61,62,63], and mitigation of oxidative stress damage [20,64,65,66,67] (Figure 1). These molecular mechanisms are elaborated in detail below.

#### 3.1.1. Protecting the Blood–Brain Barrier by Inhibiting the TGF-β1/MMP-9 Axis

Taking cardiopulmonary bypass (CPB) as an example, the implementation of hypothermia during surgery effectively reduces cerebral metabolic demand and mitigates ischemic-hypoxic injury [68]. As a highly oxygen-dependent organ, the brain is particularly vulnerable during CPB to thromboembolism, ischemia–reperfusion injury, and systemic inflammatory responses, all of which significantly increase the risk of postoperative neurological dysfunction [68,69,70]. Controlled hypothermia combined with external cooling can inhibit neuronal apoptosis and reduce secondary neurological damage [68].

Experimental data indicate that regulating rat core body temperature to 32 °C during CPB significantly upregulates intracellular CIRP expression in brain tissue [14]. Overexpression of iCIRP downregulates transforming growth factor-β1 (TGF-β1) expression [14]. As a pro-fibrotic factor, TGF-β1 induces pathological deposition of extracellular matrix components, increases endothelial permeability, and disrupts tight junction proteins in the blood–brain barrier (BBB) by activating matrix metalloproteinase-9 (MMP-9) [14]. This ultimately leads to inflammatory cell infiltration and facilitates the penetration of inflammatory mediators, such as tumor necrosis factor-α (TNF-α), and the oxidative stress mediator malondialdehyde (MDA) into brain tissue [14]. These mechanisms elucidate how subhypothermia therapy maintains BBB structural integrity through the iCIRP/TGF-β1 regulatory axis, effectively mitigating the progression of secondary neurological damage. Notably, the neuroprotective effects of CIRP appear independent of direct ATP-dependent metabolic suppression, suggesting that it may exert biological effects through non-metabolism-dependent mechanisms [71].

#### 3.1.2. Anti-Apoptotic Mechanisms

Hypothermia therapy also exerts neuroprotective effects by inhibiting the activation of apoptotic signaling pathways. Studies have also shown that iCIRP preserves the stemness of neural stem cells, as demonstrated by Saito et al. [15]. Experimental data indicate that under conditions of epidermal growth factor (EGF) deficiency, hypothermia therapy significantly enhances the survival rate of MEB5 mouse neural stem cells by upregulating intracellular CIRP expression, suggesting that iCIRP possesses anti-apoptotic functions [15].

iCIRP antagonizes neuronal apoptosis through multiple pathways. First, it inhibits hypoxia-induced apoptotic cascades by destabilizing hypoxia-inducible factor-1α (HIF-1α), a process likely closely linked to the activation of the microRNA-23a/CIRP signaling pathway under hypoxic conditions [16,17]. Second, iCIRP promotes neuronal survival by activating the extracellular signal-regulated kinase (ERK) and protein kinase B (AKT) signaling pathways [18,19,20]. In a mouse embryonic fibroblast (MEF) apoptosis model induced by tumor necrosis factor-α (TNF-α), iCIRP activates the ERK pathway to inhibit apoptosis [18]. In TBI models, mild hypothermia at 31 ± 0.5 °C significantly enhances CIRP transcription and translation levels in the cortical, hippocampal, and hypothalamic regions, with the most pronounced upregulation observed in the hypothalamus [19]. By activating the extracellular signal-regulated kinase 1/2 (ERK1/2) signaling pathway, iCIRP counteracts programmed neuronal death, thereby exerting specific neuroprotective effects [19]. In hydrogen peroxide (H_2_O_2_)-induced neuronal apoptosis models, iCIRP confers neuroprotection under oxidative stress by simultaneously activating the AKT and ERK signaling pathways [20].

Furthermore, iCIRP inhibits mitochondrial apoptosis pathways, thereby enhancing neuronal survival [61,62,63]. Studies on cardiac arrest have shown that hypothermia-induced iCIRP alleviates mitochondrial calcium overload by reducing the expression of the calcium transporters inositol 1,4,5-trisphosphate receptor (IP3R) and voltage-dependent anion channel 1 (VDAC1) on the mitochondrial-associated endoplasmic reticulum membrane (MAM) [62]. This restoration of ATP synthesis capacity ultimately improves neuronal survival and mitigates secondary brain injury caused by cardiac arrest [62]. Additionally, iCIRP directly suppresses the production of mitochondrial apoptosis-inducing factors, exerting significant neuroprotective effects during cardiac arrest [63].

#### 3.1.3. Counteracting Oxidative Stress Damage

Hypothermia-induced iCIRP exerts neuroprotective effects by mitigating oxidative stress damage through two primary mechanisms. First, as previously described, it inhibits oxidative stress-induced neuronal apoptosis by activating the Akt and ERK signaling pathways [20]. Second, it suppresses intracellular oxygen radical production by upregulating thioredoxin (TRX) expression [64,65,66,67]. The burst of reactive oxygen species (ROS) triggered by oxidative stress is a central pathological mechanism that initiates programmed neuronal death [72]. As a key component of ROS, excessive oxygen radical production mediates lipid oxidation (e.g., malondialdehyde (MDA) formation), protein damage, and DNA fragmentation, directly impairing neuronal structure and function [73,74,75,76]. Hypothermia-induced iCIRP can be phosphorylated by glycogen synthase kinase 3β (GSK3β), translocated to the cytoplasm, and bound to the 3′ UTR of TRX transcripts, thereby promoting TRX translation and increasing its protein expression [66]. This mechanism has been validated in a UV-induced oxidative stress model using human colorectal cancer RKO cells [66]. As a potent antioxidant, TRX exerts cytoprotective effects by reducing disulfide bonds and scavenging oxygen-free radicals, counteracting oxidative stress-induced cellular damage [67].

Early studies demonstrated that both ischemia–reperfusion injury and exogenous H_2_O_2_ stimulation significantly suppressed CIRP mRNA expression in neurons, suggesting that ischemia–reperfusion may negatively regulate CIRP via oxidative stress, thereby exacerbating neuronal damage [77]. This indirectly confirms the neuroprotective role of iCIRP under oxidative stress conditions. Although subsequent studies have supported the inductive effect of ischemia–reperfusion injury on CIRP expression [13,78,79], given the central role of oxidative stress in cerebral ischemia–reperfusion injury, targeting the CIRP signaling axis may offer novel intervention strategies for neuroprotection.

### 3.2. Controversy and Temperature-Dependent Effects

It is noteworthy that the biological effects of CIRP in hypothermia therapy remain a subject of academic debate. A study using the subarachnoid hemorrhage (SAH) model by Haibin Dai et al. has demonstrated that mild hypothermic intervention at 30–31 °C can suppress inflammatory signaling and mitigate early secondary brain injury following SAH by downregulating CIRP expression in temporal cortex neurons [80].

In terms of the regulatory mechanism, toxic substances such as heme [81,82] and ROS [83,84] released during hematoma breakdown after SAH may drive the transcription of CIRP mRNA and protein synthesis through unknown downstream transcription factors, resulting in the peak expression level of CIRP one day post-injury [80]. However, there is currently no direct experimental evidence to confirm that heme/ROS can specifically activate CIRP-related transcription factors in the SAH model, nor have the specific transcription regulatory molecules involved been identified. This association is merely a reasonable speculation.

Early studies have confirmed that mild hypothermia can reduce the cerebral metabolic rate [85,86], suppressing ROS production and enhancing ROS scavenging efficiency [87,88]. Additionally, Haibin Dai et al. detected via RT-PCR that mild hypothermia maintained at 30–31 °C for 6 h can significantly decrease the CIRP mRNA levels in the SAH model at 1 d, 3 d, and 7 d [80]. Based on this indirect evidence, it is suggested that hypothermia may inhibit the abnormal elevation of CIRP through transcriptional regulation. However, due to the lack of direct verification experiments such as transcription factor binding activity assay and chromatin immunoprecipitation (ChIP), the possibility that this effect originates from post-translational mechanisms such as mRNA stability regulation cannot be completely ruled out, and the direct regulatory effect of hypothermia on CIRP transcription still lacks experimental confirmation.

Given the functional difference of CIRP, protective intracellularly and damaging extracellularly, we also propose a hypothesis: hypothermia may stabilize the integrity of the nuclear membrane structure, reduce the nucleocytoplasmic translocation of CIRP, and decrease its passive release mediated by damaged cell membranes. This, in turn, lowers the extracellular accumulation of eCIRP and attenuates the amplification of downstream inflammatory cascades. However, this hypothesis lacks direct experimental support for the association between nuclear membrane stability detection and CIRP translocation, and whether nuclear membrane stability is involved in regulating the nucleocytoplasmic translocation of CIRP requires further verification.

Furthermore, the study by Haibin Dai et al. has directly confirmed through multi-dimensional experiments that the high expression of CIRP under SAH pathological conditions is closely associated with abnormal activation of the mitochondrial apoptotic pathway. In the brain tissue of rats in the SAH group, the expression of pro-apoptotic molecules Bax and caspase-3/9 was significantly upregulated, the expression of anti-apoptotic molecule Bcl-2 was decreased, the release of cytochrome c from mitochondria into the cytoplasm was increased, and the mitochondria exhibited structural damage such as cristae disruption and vacuolization [80]. Based on this clear association, it is speculated that hypothermia may indirectly enhance the neuroprotective effect mediated by CIRP downregulation by directly inhibiting the excessive activation of this apoptotic pathway and blocking the potential association among CIRP overexpression, mitochondrial damage, and neuronal apoptosis [80].

The core mechanism underlying the aforementioned regulatory differences may be attributed to the synergistic effect of the disease-specific pathological microenvironment and CIRP expression levels. During the pathological process of SAH, CIRP often exhibits “pathological overexpression” and primarily exerts pro-inflammatory and damaging effects in the form of eCIRP [80]. At this stage, the primary role of hypothermic intervention may be to inhibit its abnormal activation [80] In contrast, in other brain injury models such as CPB and TBI, the baseline expression level of CIRP is relatively low, without significant overactivation after injury, and mild hypothermia may exert neuroprotective effects by upregulating the expression of intracellular CIRP (iCIRP) and leveraging its intracellular biological functions, including anti-apoptosis and maintenance of blood–brain barrier integrity [14,19].

However, when body temperature drops below therapeutic ranges, severe hypothermia itself becomes a potent stressor, triggering cell necrosis and the direct extracellular release of CIRP. eCIRP then activates the bromodomain-containing protein 2 (BRD2)/NF-κB signaling pathway, polarizing microglia toward a proinflammatory phenotype [89]. This activation leads to the massive release of proinflammatory mediators such as interleukin-5 (IL-5), interleukin-13 (IL-13), and TNF-α, ultimately exacerbating neuronal injury [89]. Since microglia function as the innate immune cells of the central nervous system, their activation state directly influences neuroinflammatory processes [25]. Collectively, these studies suggest that the dual neuroprotective and neurotoxic effects of CIRP are dependent on temperature and spatial context. Therefore, precisely defining the therapeutic temperature window and pathological conditions is essential for its potential clinical application.

## 4. Overview of the Core Pathological Mechanisms of CIRP in Neurological Disorders

As previously discussed, intracellular CIRP functions as an RNA-binding protein under physiological conditions, maintaining normal circadian rhythms in the nervous system by regulating RNA stability and translation efficiency [31]. It also exerts neuroprotective effects during cold therapy [14,15,18,19,20,61,62,63,64,65,66,67]. However, following neural injury, CIRP is released extracellularly (eCIRP) and acts as a DAMP, mediating inflammation and tissue damage. The concept of eCIRP acting as a DAMP was established by the seminal work of Qiang et al. (2013) [9]. This pathological role has been demonstrated in various neurological disorders, including IS [21,22,23,90], cerebral ischemia–reperfusion (I/R) injury [24], ICH [26], traumatic brain injury (TBI) [25], and Alzheimer’s disease (AD) [27,91].

Mechanistic analyses across multiple studies indicate that eCIRP’s pathological effects follow a three-tiered process: release triggering, signal initiation, and effect differentiation. The eCIRP-TLR4 signaling axis serves as the central hub, although eCIRP may also exert effects through non-TLR4-dependent pathways. Disease-specific microenvironments further influence downstream effect differentiation, establishing an regulation model pathways with disease microenvironments. This model provides a unified framework for understanding the neuropathological effects of eCIRP.

### 4.1. Release Trigger Mechanism of eCIRP

Despite significant heterogeneity in the initial pathological triggers of various neurological disorders—IS and cerebral I/R injury primarily involve hypoxic–ischemic mechanisms [21,22]; ICH is associated with hematoma formation [92]; traumatic brain injury (TBI) results from mechanical disruption of the blood–brain barrier [93]; and AD and chronic alcohol-related brain injury are driven by prolonged pathological stimuli such as Aβ deposition and ethanol exposure [94,95,96,97]—current research consensus indicates that eCIRP formation represents a common molecular event during the initiation phase of these pathological processes. Experimental evidence shows that extracellular release of CIRP occurs via two distinct patterns: acute passive release and chronic active secretion. The acute passive release pattern is predominantly observed in sudden-onset brain injuries such as IS and TBI. This release is characterized by rapid initiation and abrupt elevation of eCIRP concentration, with significant increases typically detectable within hours of injury onset [25]. Moreover, eCIRP levels positively correlate with injury severity; for example, in IS, eCIRP concentrations correlate with infarct volume and NIH Stroke Scale (NIHSS) scores [90], suggesting its potential as an early biomarker for acute brain injury. In contrast, in chronic progressive neurological diseases such as alcohol-related brain injury and AD, eCIRP release appears to follow a chronic active secretion pattern, characterized by sustained release and gradual increases in concentration as disease pathology advances. However, current research in this area has notable limitations: most studies focus on elevated CIRP expression in acute alcohol exposure scenarios [27,95], while investigations into the dynamic changes and regulatory mechanisms of eCIRP during chronic alcohol exposure remain scarce.

### 4.2. Initiation of eCIRP Signaling

The eCIRP-TLR4 signaling axis constitutes the central pathway through which eCIRP mediates its pathological effects in neurological disorders. Studies have confirmed, via surface plasmon resonance (SPR) experiments, that eCIRP is a specific ligand for Toll-like receptor 4 (TLR4), exhibiting dissociation constants (Kd) of approximately 6.17 × 10^−7^ mol/L [9,10]. Their binding can be competitively inhibited by antagonists such as the C23 peptide [23,98]. Upon TLR4 activation, signaling proceeds through two adaptor protein-dependent branches. The first is the myeloid differentiation factor 88 (MyD88)-dependent branch, which recruits interleukin-1 receptor-associated kinase (IRAK) family kinases via MyD88, subsequently activating the NF-κB and mitogen-activated protein kinase (MAPK) (p38 mitogen-activated protein kinase (p38), c-Jun N-terminal kinase (JNK)) pathways [99,100,101]. This branch primarily regulates the transcriptional release of proinflammatory cytokines, including TNF-α, interleukin 1β (IL-1β), and interleukin 6 (IL-6) [99,100,101]. In diseases characterized by acute inflammation, such as ischemic stroke and ICH, this pathway serves as the principal activation route and a key component in inflammatory amplification [22,26,102]. The second branch is TIR-domain-containing adapter-inducing interferon-β (TRIF)-dependent, recruiting TNF receptor-associated factor 3 (TRAF3) and TANK binding kinase 1 (TBK1) via TRIF to activate interferon regulatory factor 3 (IRF3), thereby promoting interferon production (e.g., interferon-β (IFN-β)) and modulating immune responses [99,103]. However, current research on eCIRP has yet to elucidate the specific effector molecules and pathological roles of this branch in neurological diseases, highlighting the need for further investigation into its precise functions.

Furthermore, in certain diseases, eCIRP may exert pathological effects through disease-specific pathways independent of the TLR4 signaling axis. For instance, in ICH, eCIRP amplifies inflammatory responses via the interleukin 6 receptor (IL-6R)/signal transducer and activator of transcription 3 (STAT3) signaling pathway [104]. In TBI, eCIRP induces neuronal apoptosis by activating the endoplasmic reticulum stress (ERS)-related protein kinase R-like endoplasmic reticulum kinase (PERK)/activating transcription factor 4 (ATF4)/DNA damage-inducible transcript 3 (CHOP) signaling cascade [25]. In AD, eCIRP promotes pathological tau phosphorylation via IL-6 receptor α (IL-6Rα)-mediated STAT3/cytokinin-dependent protein kinase 5 (Cdk5) signaling [27], among others. Detailed mechanisms will be elaborated in subsequent sections.

### 4.3. Common and Distinct Downstream Effects: Core Phenotypes of Inflammatory Amplification and Neuronal Injury

In all diseases, the final effects mediated by eCIRP converge on inflammatory responses and neuronal damage, specific manifestations vary significantly due to differences in signaling pathway selection and the influence of the disease microenvironment. Common features include inflammatory amplification, primarily driven by sustained NF-κB activation [22,24,26,102]. Neuronal injury presents as apoptosis (Cysteine aspartic protease (Caspase-3)-mediated) [25], pyroptosis (NOD-like receptor thermal protein domain associated protein 3 (NLRP3)-Cysteine aspartic protease (Caspase-1)-Gasdermin D (GSDMD)-mediated) [24,27], or necrosis [27]. Distinct effects include, in IS, additional neutrophil extracellular trap (NET) activation leading to blood–brain barrier disruption [21]; in AD, it modulates Aβ/tau pathology [27,91,94,105,106,107]; and in ICH, it may synergistically amplify inflammation with hemoglobin [26,108]. These observations reflect a regulatory model based on the interplay between signaling pathways and the disease microenvironment.

## 5. The Role of CIRP in Various Neurological Disorders

### 5.1. Ischemic Stroke (IS)

**Figure 2:**
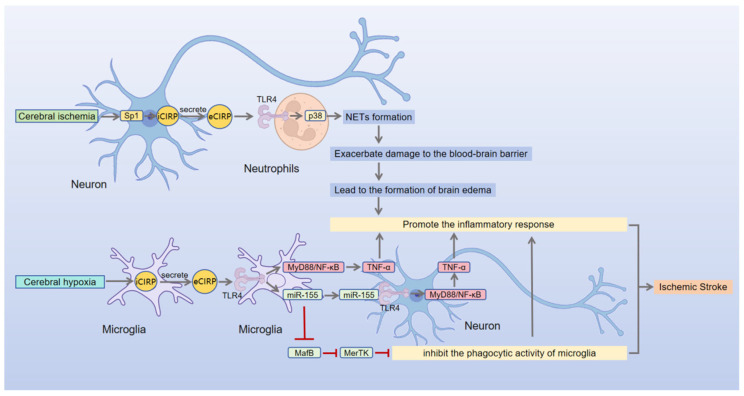
The role of CIRP in ischemic stroke. During ischemic stroke, ischemia and hypoxia induce the overexpression and release of CIRP in neurons and microglia. eCIRP promotes NETs formation via the TLR4/p38 pathway while simultaneously enhancing the release of inflammatory cytokines through the TLR4/MyD88/NF-κB pathway. Subsequently, eCIRP induces microglial miR-155 expression, which amplifies inflammation by activating TLR4/MyD88 and inhibiting the MafB-MerTK axis. Sp1: transcription factor sp1; TLR4: Toll-like receptor 4; p38: p38 mitogen-activated protein kinase; NETs: neutrophil extracellular trapping networks; MyD88: myeloid differentiation factor 88; NF-κB: nuclear factor-κB; miR-155: microRNA-155; MafB: MAF bZIP transcription factor B. MerTK: MER proto-oncogene, tyrosine kinase.

Stroke is one of the leading causes of disability and death worldwide, with its incidence showing a significant upward trend in developing countries, predominantly driven by ischemic stroke (IS) caused by arterial occlusion [109]. Disruption of the blood–brain barrier is a key pathophysiological feature of IS, directly contributing to the progression of secondary brain injury [110]. eCIRP exacerbates brain injury in IS through multiple mechanisms, with the TLR4 signaling axis serving as a central pathway (Figure 2). Zhou et al. (2014) provided the first evidence that eCIRP promotes neuroinflammation in ischemic stroke by activating the TLR4/NF-κB pathway [22]. More recent studies have expanded on this.

First, during the injury initiation phase, ischemia triggers the neuronal transcription factor Sp1 to mediate CIRP transcription activation and sustained extracellular release [21]. Neuron-derived eCIRP induces neutrophils to release components such as chromatin and elastase for NET formation via the TLR4/p38-MAPK signaling axis [21]. The formed NETs indirectly degrade tight junction proteins of brain endothelial cells (e.g., ZO-1, Claudin-5, Occludin) by releasing cytotoxic components, including elastase and peroxidase, thereby contributing to blood–brain barrier disruption and vasogenic cerebral edema formation [21,111]. Concurrently, hypoxia-induced microglial eCIRP activates TLR4/MyD88-dependent NF-κB pathways, driving the expression of proinflammatory cytokines (e.g., TNF-α) and triggering neuronal injury and apoptosis [22]. It should be noted that current studies have not clarified whether the NETs responsible for brain endothelial barrier injury are mainly derived from intravascular or parenchymal neutrophils. It is speculated that parenchymal NETs (consistent with the peak time of barrier injury) may be the main contributor, while early-formed intravascular NETs may also play a partial role [21].

Subsequently, during the amplification and effector phases of inflammation, eCIRP induces the expression of proinflammatory microRNA-155 (miR-155) in microglia via the TLR4 pathway [23]. In IS, miR-155 exacerbates injury through two mechanisms. First, by activating the TLR4/MyD88 pathway, thereby contributing to cellular damage [102]. Second, by suppressing the pro-phagocytic transcription factor MAF bZIP (MafB), which reduces MER proto-oncogene, tyrosine kinase (MerTK) expression on the microglial surface and inhibits phagocytic function [23]. This ultimately impedes the resolution of neuroinflammation and delays stroke recovery [112]. And the CIRP-derived peptide segment C23 can mitigate miR-155 induction by blocking eCIRP-TLR4 interactions, thereby restoring MerTK expression and microglial phagocytosis, which ultimately improves outcomes in transient middle cerebral artery occlusion (tMCAO) mouse models [23]. These findings provide experimental evidence supporting targeted intervention against eCIRP.

Furthermore, given the critical roles of astrocytes and endothelial cells in maintaining the blood–brain barrier [113,114], as well as the interactions between astrocytes and microglia under pathological conditions [115,116], it is speculated that eCIRP may further exacerbate blood–brain barrier disruption and neuronal injury during the pathological progression of IS by directly or indirectly regulating astrocyte polarization and function. However, direct evidence supporting this mechanism is currently lacking and warrants further investigation.

In summary, the evidence presented systematically elucidates the multifaceted mechanisms by which eCIRP functions as a central mediator in the pathological development of IS, confirming its pivotal role in the disease process (Figure 2). A clinical cohort study involving 148 patients with acute IS has further demonstrated that serum eCIRP levels increase significantly in the early stage of the disease and gradually decrease after 3 days [90]. Early serum eCIRP levels are significantly positively correlated with cerebral infarction volume (Spearman’s correlation coefficient r = 0.240, *p* < 0.01, coefficient of determination R^2^ = 0.058), NIHSS scores (r = 0.304, *p* < 0.01, R^2^ = 0.092), and modified Rankin Scale (mRS) functional outcome scores at 90 days post-onset (r = 0.204, *p* < 0.05, R^2^ = 0.041) [90]. Furthermore, the area under the receiver operating characteristic (ROC) curve (AUC) for eCIRP in predicting acute IS is 0.654 (95% confidence interval [CI] = 0.562–0.746, *p* < 0.001), with an optimal cutoff value of 303.24 pg/mL, a specificity of 76.7%, and a sensitivity of 51.6% [90]. These findings suggest that eCIRP has dual value as both a therapeutic target for modulating pathological mechanisms and a clinical biomarker of disease severity in IS.

### 5.2. Cerebral Ischemia–Reperfusion (I/R) Injury

Rapid recanalization of occluded arteries through pharmacologic thrombolysis or mechanical thrombectomy is the primary therapeutic approach for IS [117,118]. Cerebral ischemia–reperfusion (I/R) injury is a common and severe complication following IS treatment, characterized by a paradoxical exacerbation of damage upon restoration of blood flow [24]. The effects of I/R on cold-inducible RNA-binding protein (CIRP) expression remain controversial: early studies suggested that cerebral I/R downregulates CIRP expression in the hippocampus via oxidative stress [77], whereas more recent research increasingly supports an I/R-induced upregulation of CIRP [13,78,79].

**Figure 3:**
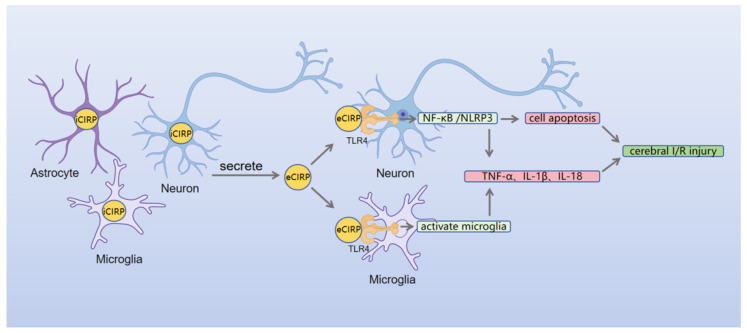
The role of CIRP in ischemic stroke. Primarily derived from neurons, eCIRP promotes neuronal apoptosis through the NF-κB/NLRP3 pathway and synergistically amplifies the inflammatory response during cerebral ischemia–reperfusion by activating microglia. NLRP3: NOD-like receptor thermal protein domain associated protein 3.

The pathological mechanism of eCIRP in cerebral I/R injury remains incompletely understood. Recent in vivo and in vitro studies have demonstrated that eCIRP mediates activation of the TLR4/NF-κB/NLRP3 signaling pathway in neurons, promoting glial cell polarization toward a pro-inflammatory phenotype and the release of serum inflammatory mediators [24]. This cascade ultimately exacerbates neuronal injury in cerebral I/R [119] (Figure 3). While this mechanism partially overlaps with the NF-κB activation pathway of eCIRP observed in ischemic stroke (IS) [22], it differs in its explicit involvement of NLRP3 inflammasomes in cerebral I/R [24]. This difference is likely due to increased oxidative stress during reperfusion and a lowered threshold for NLRP3 inflammasome activation.

### 5.3. Intracerebral Hemorrhage (ICH)

Intracerebral hemorrhage (ICH), a subtype of hemorrhagic stroke caused by the spontaneous rupture of intracerebral blood vessels, is pathologically characterized by hematoma formation resulting from microvascular structural disruption in deep brain regions [92]. Neuroinflammation plays a central role in the secondary brain injury associated with ICH [120]. Recent studies have confirmed that eCIRP, a DAMP, contributes to the pathological process of ICH through the TLR4/NF-κB and IL-6R/STAT3 signaling pathways, highlighting its dual potential as both a biomarker and a therapeutic target [26,104].

In ICH models, CIRP mRNA and protein levels in brain tissue surrounding the hematoma significantly increased 1–3 days post-injury, with no notable elevation observed at 5–7 days, indicating a time-dependent pattern [26]. CIRP knockout mice exhibited significantly fewer degenerating neurons and reduced levels of inflammatory mediators (IL-6, TNF-α, IL-1β) in the perivascular area on day 3 post-ICH compared to wild-type mice [26]. Additionally, brain water content (BWC) and neurological deficit scores (NDS) showed sustained improvement from days 3 to 7, directly confirming CIRP’s role in exacerbating ICH injury [26].

**Figure 4:**
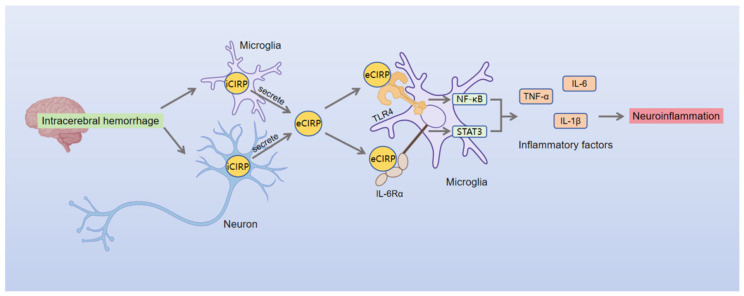
The role of CIRP in intracerebral hemorrhage. Intracerebral hemorrhage induces the overexpression and release of CIRP from neuron and microglia. eCIRP upregulates inflammatory factors such as TNF-α, IL-1β, and IL-6 through the TLR4/NF-κB pathway and IL-6R/STAT3 pathway in microglia, thereby triggering neuroinflammation. IL-6Rα: Interleukin 6 receptor α; STAT3: Signal transducer and activator of transcription 3.

Mechanistic studies reveal eCIRP exerts pathological effects through two distinct pathways (Figure 4). First, via the TLR4/NF-κB signaling pathway: CIRP knockout significantly reduces TLR4 expression and NF-κB levels in perivascular brain tissue at day 3 post-ICH [26], suggesting that eCIRP acts as a TLR4 ligand to activate the NF-κB signaling cascade, thereby contributing to secondary neuroinflammatory injury after ICH. Given that heme independently activates the TLR4/NF-κB pathway to exacerbate ICH-related inflammation [108], and considering the potential functional overlap and co-localization of eCIRP with the lesion microenvironment in ICH, a synergistic effect between the two in activating the TLR4/NF-κB pathway is hypothesized, although direct evidence of their interaction is currently lacking. Second, the IL-6R/STAT3 signaling pathway is involved. Neuron-derived eCIRP binds to the IL-6 receptor (IL-6R) on the surface of microglia, activating the downstream STAT3 signaling pathway [104]. This activation drives microglia toward a pro-inflammatory phenotype and promotes the release of inflammatory cytokines [104].

Regarding therapeutic potential and clinical relevance, the peptide inhibitor Tat-CIRP-CMA (TCC), which targets the eCIRP-IL-6R interaction, demonstrated clear efficacy. In both in vitro oxygen-glucose deprivation (OGD) models and rat ICH models, TCC significantly reduced neuronal CIRP release, inhibited microglial activation and IL-6R/STAT3 pathway activation, enhanced microglial phagocytic function, and decreased inflammatory cytokine levels [104]. These findings provide new experimental evidence and translational directions for precision intervention in ICH. Furthermore, clinical analysis revealed a significant positive correlation between peripheral blood CIRP expression levels and infarct volume in 51 human ICH patients compared with 37 age-matched healthy controls (R^2^ = 0.6902, *p* < 0.0001), suggesting its potential as a biomarker for assessing ICH severity and prognosis [104].

### 5.4. Traumatic Brain Injury (TBI)

**Figure 5:**
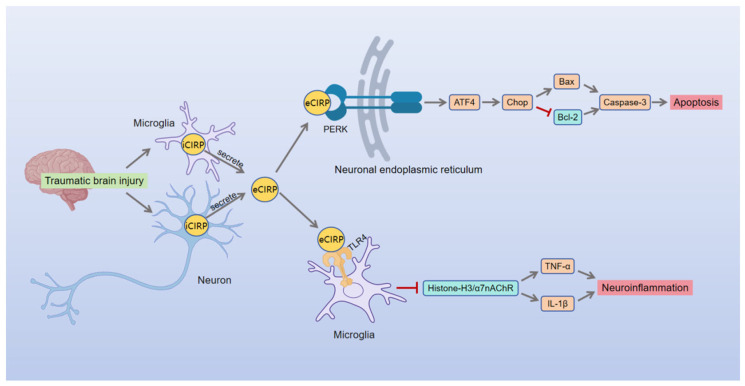
The role of CIRP in traumatic brain injury. Traumatic brain injury induces the overexpression and release of CIRP in neurons and microglia. eCIRP promotes neuronal apoptosis through the PERK/ATF4/CHOP pathway while triggering neuroinflammation via the TLR4/histone H3/α7nAChR pathway. PERK: protein kinase R-like endoplasmic reticulum kinase; ATF4: activating transcription factor 4; Chop: DNA damage-inducible transcript 3; Bax: B lymphoblastoma-2 gene-associated X protein; Bcl-2: B lymphoblastoma-2 gene; Caspase-3: cysteine aspartic protease-3; α7nAChR: nicotinic acetylcholine receptor α7 subunit; IL-1β: interleukin-1β.

Traumatic brain injury (TBI) is a brain dysfunction or structural damage caused by mechanical force [93], with an estimated annual global incidence of approximately 69 million cases, representing a significant public health burden [25]. In TBI, eCIRP not only activates TLR4-dependent pathways but also mediates non-TLR4-dependent endoplasmic reticulum stress (ERS)-associated apoptotic pathways [25] (Figure 5).

Specifically, in TBI models, CIRP mRNA levels in injured brain regions increase within 4 h post-injury, peak at 1 day, and sustain protein expression for at least 7 days. This rapid release may be directly induced by mechanical forces [25]. Functionally, eCIRP exacerbates TBI injury through two independent pathways (Figure 5). First, by triggering a TLR4-mediated cascade involving histone H3/α7 nicotinic acetylcholine receptor (α7nAChR) signaling, which drives phenotypic shifts toward activation in microglia and astrocytes, thereby intensifying the neuroinflammatory microenvironment [25]. Second, eCIRP significantly promotes neuronal apoptosis by activating the ERS-associated PERK/ATF4/CHOP signaling cascade [25].

Furthermore, CIRP knockout significantly reduces brain volume loss and apoptotic cell counts in TBI mice [25], suggesting that CIRP is a key mediator of secondary TBI injury. These findings establish eCIRP as a pivotal regulator in secondary TBI injury. It not only serves as a potential therapeutic target for modulating neuronal apoptosis and glial hyperactivation but also functions as a critical biomarker for assessing the severity of brain injury during TBI.

### 5.5. Alzheimer’s Disease (AD)

**Figure 6:**
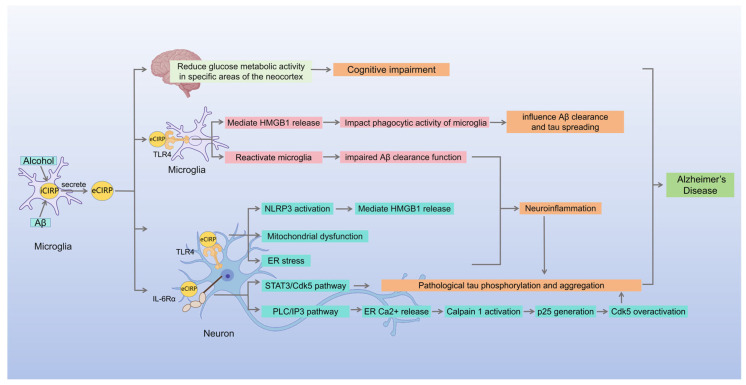
The role of CIRP in mediating alcohol related Alzheimer’s disease. IL-6Rα: interleukin-6 receptor α; STAT3: signal transducer and activator of transcription 3; Cdk5: cytokinin-dependent protein kinase 5; NLRP3: nucleotide-binding oligomerization structural domain-like receptor protein 3; HMGB1: high mobility group protein B1; PLC: phospholipase C; IP3: Inositol 1,4,5-trisphosphate; ER: endoplasmic reticulum.

Alzheimer’s disease (AD) is a leading cause of cognitive decline, characterized by pathological hallmarks such as β-amyloid (Aβ) deposition, abnormal tau protein phosphorylation, and neurofibrillary tangles [94]. Chronic ethanol exposure is a significant risk factor for AD [121,122,123,124]. eCIRP contributes to AD progression by modulating neuroinflammation and pathological protein accumulation, potentially serving as a molecular link between alcohol exposure and AD pathology (Figure 6).

In the pathological microenvironment of AD, microglia serve as a major source of eCIRP release [27,105,125,126]. The eCIRP they secrete can exacerbate disease progression through inflammatory amplification mechanisms [27,105,125,126]. Specifically, Aβ-mediated neuronal stress associated with AD significantly increases eCIRP release from BV2 microglia in a time-dependent manner, reaching 3.2 times the control level after 24 h [125]. Similarly, ethanol exposure induces transcriptional activation and extracellular release of CIRP in microglia, establishing microglia as a key source of eCIRP in alcohol-associated AD [126].

eCIRP regulates neuroinflammation and pathological protein dynamics in AD through multiple mechanisms. First, eCIRP promotes pathological phosphorylation of tau protein via interleukin-6 receptor α (IL-6Rα)-mediated STAT3/Cdk5 signaling [27,125]. Further studies confirm that eCIRP triggers Ca^2+^ release from endoplasmic reticulum (ER) calcium stores through an IL-6Rα/phospholipase C (PLC)/Inositol 1,4,5-trisphosphate (IP3)-dependent pathway, which elevates intracellular free Ca^2+^ concentration [105]. This Ca^2+^ elevation specifically activates calpain 1 while reducing the expression of its endogenous inhibitor calpastatin [105]. This dual mechanism promotes calpain 1-mediated hydrolysis of the Cdk5 regulator p35, generating the neurotoxic fragment p25, which ultimately leads to excessive Cdk5 activation and exacerbated tau phosphorylation [105]. The peptide C23 has been demonstrated to specifically block the binding of eCIRP to IL-6Rα, thereby inhibiting the IL-6Rα/STAT3/Cdk5 signaling pathway [125]. Additionally, C23 significantly attenuates eCIRP-induced p25 generation by inhibiting ER calcium release and calpain 1 activation mediated by the IL-6Rα/PLC/IP3 axis [105], suggesting its translational potential for eCIRP-targeted AD therapies. Second, eCIRP mediates various injury pathways via TLR4, including neuronal endoplasmic reticulum stress, NLRP3 inflammasome activation, mitochondrial dysfunction, and microglial activation, ultimately triggering neuroinflammation, neuronal injury, and even cell death [27]. A pathological link has been established between neuroinflammation and abnormal tau phosphorylation [106]. And activation of the inflammatory phenotype in microglia is associated with impaired Aβ clearance capacity [107]. Additionally, eCIRP may regulate Aβ metabolic balance and tau protein propagation by modulating high-mobility group box 1 (HMGB1) release pathways and microglial phagocytic activity [27]. Furthermore, CIRP overexpression in astrocytes suppresses intracellular urokinase-type plasminogen activator (uPA) expression, thereby promoting neuronal Aβ1-42 production and tau phosphorylation, ultimately increasing AD risk [91].

Alcohol consumption is an independent risk factor for neurological disorders, inducing multisystemic toxic effects and being associated with various brain pathologies, including hippocampal neuronal damage [96,97]. It particularly affects central nervous system regions involved in memory encoding, motor coordination, and emotional regulation [127,128], and has been confirmed as a significant risk factor for AD [121,122,123,124]. eCIRP serves as a key mediator of alcohol-induced metabolic dysfunction and cognitive impairment in localized brain regions [95]. Specifically, ethanol exposure induces transcriptional activation of CIRP in microglia, followed by its extracellular release, which triggers pro-inflammatory factors such as TNF-α and IL-1β, while causing abnormal elevations in serum markers of tissue injury, including aspartate aminotransferase (AST), alanine aminotransferase (ALT), and lactate dehydrogenase (LDH) [126]. This chronic inflammation not only directly reduces glucose metabolism in neocortical memory-related regions (e.g., the temporal association cortex), impairing object location memory [95], but also promotes Aβ deposition and tau phosphorylation through sustained stress [27,91,106,107], thereby creating conditions conducive to the initiation of AD pathology. Behavioral studies indicate that silencing CIRP expression partially ameliorates alcohol-induced cognitive impairment [95] and reduces proinflammatory factor levels [126], suggesting that eCIRP may serve as a therapeutic target for alcohol-related AD pathology.

Although the multi-target regulatory network of eCIRP in AD pathology remains incompletely understood, molecular intervention strategies targeting CIRP may offer novel therapeutic avenues for AD-related pathological processes, particularly alcohol-associated AD.

### 5.6. Cross-Disease Mechanism Summary and Therapeutic Implications of eCIRP in Neurological Disorders

The pathological role of eCIRP in various neurological disorders adheres to the universal principle of TLR4 serving as the central hub, with neuroinflammation and neuronal injury as common outcomes. However, it also displays distinct disease-specific characteristics influenced by variations in the microenvironment. The common and specific mechanisms are summarized in Table 1.

The disease-specific differentiation of the CIRP pathway likely arises from the selective activation of signaling axes by the pathological microenvironment. Acute injuries—such as IS, TBI, and ICH—are characterized by rapid cellular disruption and the transient release of toxic substances within the microenvironment. Neurons and microglia, the brain’s primary functional cells, rapidly release eCIRP to initiate inflammatory responses [21,25,26,104]. The hypoxic–ischemic and vascular injury microenvironment in ischemic stroke preferentially activates NET-related pathways via eCIRP, mediating blood–brain barrier disruption [21]. Mechanical stress in TBI triggers endoplasmic reticulum stress pathways, primarily inducing neuronal apoptosis [25]. The heme-accumulating microenvironment in ICH may synergize with eCIRP to amplify TLR4-mediated inflammatory cascades [108]. Conversely, the microenvironment of the chronic degenerative disease AD is characterized by the persistent accumulation of Aβ/tau pathological proteins [94]. In AD, microglia continuously release eCIRP to sustain chronic inflammation [27,105,125,126], while astrocytes regulate pathological protein metabolism through intracellular CIRP overexpression [91], forming a vicious cycle of inflammation and protein accumulation. This contrasts sharply with the acute injury-specific inflammation and structural disruption. In cerebral ischemia–reperfusion, the reperfusion stress microenvironment lowers the activation threshold of the NLRP3 inflammasome via oxidative stress [24,119], promoting cross-activation between the eCIRP-TLR4 pathway and inflammasomes. This interaction amplifies synergistic injury effects resulting from proinflammatory factor release and calcium overload.

Optimizing therapeutic strategies requires leveraging the universality of shared mechanisms while implementing individualized adjustments based on disease-specific pathways and microenvironmental characteristics. Interventions targeting shared mechanisms have demonstrated cross-disease applicability in multiple studies, with two classes of peptide inhibitors showing particularly promising translational potential. First, the TLR4 antagonist C23 peptide competitively blocks eCIRP-TLR4 interactions [9]. In an IS tMCAO mouse model, it has been demonstrated to suppress inflammatory amplification and neuronal injury [23]. Additionally, C23 simultaneously targets the eCIRP-IL-6Rα binding site to inhibit the IL-6Rα/STAT3/Cdk5 and IL-6Rα/PLC/IP3 signaling pathways, revealing multi-target intervention potential in AD-related mechanisms [105,125]. Given that C23’ s targets encompass core eCIRP-mediated inflammatory pathways, it may theoretically exert broad-spectrum anti-inflammatory effects across multiple neurological disorders, including cerebral IR injury, ICH, and TBI. Second, the dual-targeted peptide inhibitor TCC simultaneously targets MD2 and IL-6Rα [104,129]. In rat ICH models, TCC has been demonstrated to reduce neuronal CIRP release, inhibit activation of the IL-6R/STAT3 pathway in microglia, and enhance microglial phagocytic function to decrease hematoma volume [104]. Considering the core mechanism in AD pathology, where eCIRP regulates tau phosphorylation via the IL-6Rα/STAT3/Cdk5 axis [27], TCC’s potential intervention in this pathway offers theoretical feasibility for delaying AD progression. Moreover, TCC’s targeting of MD2 has been validated in perioperative neurocognitive disorder (PND) models [129], suggesting its potential broad-spectrum anti-inflammatory effects across multiple neurological disorders. This hypothesis warrants further experimental validation. Furthermore, other TLR4 inhibitors, such as E5531 and Eritoran, can modulate core inflammatory pathways in most diseases by reducing NF-κB-mediated TNF-α and IL-1β release [130]. Calpain inhibitors like SNJ-1945 can intervene in calcium-dependent p25 generation in AD and alleviate cytoskeletal disruption in IS, demonstrating potential for multi-disease applications [105,131,132].

Personalized therapy requires precise targeting of disease-specific mechanisms. In IS, additional targeting of NET formation—such as with elastase inhibitors—or blood–brain barrier repair using tight junction protein stabilizers is necessary [21,111]. Early intervention with the C23 peptide can be combined to block the eCIRP-TLR4 axis [23]. For ICH, targeting the IL-6Rα/STAT3 pathway with TCC may be prioritized, potentially combined with heme scavengers (e.g., hemopexin) and iron chelators (e.g., deferoxamine) to mitigate heme-related neurotoxicity and inhibit synergistic inflammation [104,108,133]. In AD, adding Aβ scavengers, such as anti-Aβ monoclonal antibodies, or tau kinase inhibitors, including Cdk5 inhibitors, can disrupt the pathological protein cycle [91,94,105,106,107]. The inhibitory effect of the C23 peptide on tau phosphorylation may serve as an adjunctive intervention [105,125]. For cerebral I/R injury, inhibition of the NLRP3 inflammasome (e.g., MCC950) is critical and can be combined with C23 peptide to block eCIRP-mediated TLR4 activation [9,24,119,134].

In summary, the extracellular proinflammatory role of CIRP represents a well-established core pathological feature across various neurological disorders. The eCIRP-TLR4 pathway functions as a universal signaling hub, while disease specificity likely arises from microenvironmental regulation leading to divergence in downstream effectors: acute injury primarily involves inflammation and structural disruption [21,25,26,104], whereas AD is characterized by inflammation and pathological protein metabolism disorders [27,94,105,125,126]. Future therapies may adopt an integrated approach that combines common targets with disease-specific modifications. Peptide inhibitors such as C23 and TCC could address core inflammatory pathways, while targeted interventions based on the cellular origins of eCIRP and microenvironmental factors (e.g., acute ischemia, chronic pathological protein accumulation, heme accumulation) could be layered on top to guide precision treatment. Additionally, existing preclinical studies indicate that eCIRP is released early across different diseases (rising within 1–3 days of IS onset and within 24 h of ICH), suggesting its potential as a universal biomarker. Monitoring serum eCIRP levels may therefore assist in assessing disease severity [90,104].

## 6. In Summary and Future Perspective

This review systematically elucidates the biphasic regulatory roles of CIRP in the physiology and pathology of the central nervous system, clarifying that its functional dichotomy—intracellular protection and extracellular damage—serves as a fundamental basis for investigating the mechanisms underlying neurological diseases and developing therapeutic strategies.

Under physiological conditions, CIRP is primarily localized in the nucleus, where intracellular CIRP (iCIRP) regulates the stability and translational efficiency of core circadian clock genes (such as CLOCK, Per, and Dbp) by binding to their 3′ untranslated regions [31]. This positions iCIRP as a key molecule in circadian rhythm regulation. Dysregulation of iCIRP may accelerate neurodegenerative diseases, including Alzheimer’s disease (AD) and Parkinson’s disease (PD) by disrupting circadian balance [37,38,55,56,57,58]. Conversely, during hypothermia intervention, iCIRP exerts neuroprotective effects through multiple mechanisms: inhibiting the TGF-β1/MMP-9 axis to maintain blood–brain barrier integrity [14]; activating ERK1/2 and Akt pathways to antagonize neuronal apoptosis [18,19,20]; and upregulating thioredoxin (TRX) to mitigate oxidative stress damage [64,65,66,67]. These mechanisms provide crucial molecular support for hypothermia therapy in conditions such as cardiopulmonary bypass (CPB) and traumatic brain injury (TBI). Although the regulation of CIRP by hypothermia remains controversial—for example, hypothermia downregulates CIRP to suppress inflammation in subarachnoid hemorrhage models [80]—its protective effects within a specific temperature window (31–34 °C) have been validated across multiple models, offering a reference for precision hypothermia therapy [14,19,20,64,65,66,67].

Under pathological conditions involving stress injury, such as ischemia and hypoxia, CIRP undergoes nucleocytoplasmic translocation mediated by its nuclear export signal (NES). Once in the cytoplasm, CIRP not only regulates the translation process but can also be released extracellularly to form eCIRP, thereby initiating downstream inflammatory cascades [3,6]. As a damage-associated molecular pattern (DAMP), eCIRP drives disease progression through distinct pathways in conditions such as ischemic stroke (IS), cerebral ischemia–reperfusion (I/R) injury, intracerebral hemorrhage (ICH), TBI, and AD. The eCIRP-TLR4 signaling axis serves as a central mediator of its pathological effects [21,22,23,24,25,26,27]. Beyond this common TLR4 signaling pathway, eCIRP exacerbates injury via disease-specific mechanisms. In ICH, it amplifies inflammation through the IL-6R/STAT3 pathway [104]. In TBI, it regulates neuronal apoptosis via the ERS-associated PERK/ATF4/CHOP signaling cascade [25]. In AD, it promotes tau phosphorylation through the IL-6Rα/STAT3/Cdk5 and IL-6Rα/PLC/IP3 signaling pathway [27,105,125], while also influencing β-amyloid (Aβ) metabolism by modulating HMGB1 release and microglial activation [91].

Preclinical studies further demonstrate that serum eCIRP shows preliminary potential as a biomarker for brain injury diseases, with its levels significantly correlating with cerebral infarct/hemorrhage volume and neurological deficit scores in IS and ICH patients [90,104]. Specifically, serum eCIRP levels in IS patients reach a peak 1–3 days after onset and gradually decrease over the following 4–7 days. Using a cutoff value of 303.24 pg/mL, the diagnostic specificity is 76.7%, the sensitivity is 51.6%, and the area under the curve (AUC) is 0.654 [90]. It should be noted that this AUC value is lower than that of established stroke biomarkers such as S100B (AUC 0.7–0.8) [135] and imaging examinations (AUC > 0.9) [136], indicating that its standalone diagnostic efficacy is limited. Therefore, serum eCIRP is more suitable as an auxiliary assessment tool rather than an independent diagnostic indicator. In ICH patients within 24 h of onset, serum eCIRP levels show a strong positive correlation with hemorrhage volume (R^2^ = 0.6902) and can effectively distinguish patients from healthy individuals [104].

eCIRP offers a significant early response advantage: it increases markedly within 1–3 days after IS onset and within 24 h after ICH onset, allowing for early assessment of the extent of brain tissue damage. Therefore, IS patients should be tested within 72 h of onset, while ICH patients are recommended to be tested within 24 h to capture critical signals [90,104]. Its clinical incremental value is primarily reflected in two scenarios: first, for early-stage patients where imaging cannot clearly delineate the lesion extent (e.g., hyperacute IS within 6 h of onset or early minimal ICH hemorrhage), eCIRP can assist in evaluating disease severity by quantifying the degree of inflammatory damage; second, for patients unable to tolerate CT or MRI examinations (e.g., those with metal implants or renal failure), it serves as an alternative assessment method to complement the limitations of clinical scoring systems [90,104]. Compared with the traditional inflammatory marker C-reactive protein (CRP), eCIRP demonstrates significantly higher diagnostic specificity, effectively reducing false-positive results caused by nonspecific inflammation, such as infection or trauma [90,104,137,138]. However, the sensitivity of serum eCIRP in IS patients is only 51.6% [90], indicating that nearly half of IS patients may yield false-negative results, which substantially limits its clinical utility as a screening tool and renders it unsuitable for standalone population screening or settings with a high risk of missed diagnoses. Moreover, although serum eCIRP correlates strongly with hemorrhage volume in ICH patients [104], hemorrhage volume can be directly measured by CT. In this context, the incremental value of eCIRP lies in its ability to simultaneously reflect the intensity of the inflammatory cascade following hemorrhage [104]. The degree of inflammation is a key factor influencing secondary brain injury, and eCIRP provides biological information beyond CT imaging to aid in assessing disease progression and prognosis.

In addition, the clinical application of eCIRP detection still faces several practical challenges. Currently, detection relies on ELISA kits, and clinical routine detection platforms have not been widely adopted [90,104]. The detection turnaround time is relatively long, making it difficult to meet the urgent decision-making needs of hyperacute stroke. Furthermore, the reagent cost is higher than that of conventional inflammatory markers, creating economic barriers to large-scale implementation. Given these factors, eCIRP should be used in combination with CRP and other markers to enhance its overall diagnostic value [90,104,137,138]. Meanwhile, technical optimization is necessary to reduce detection costs and shorten turnaround times to improve its clinical utility.

At the therapeutic exploration level, intervention strategies based on CIRP’s biphasic characteristics—namely, “intracellular protection and extracellular injury”—have demonstrated clear translational potential. Among these, hypothermia therapy, which leverages the neuroprotective effects of iCIRP, has been clinically applied in CPB and hypoxic–ischemic encephalopathy [68,139,140]. In targeted interventions for eCIRP, the physical barrier of the blood–brain barrier (BBB) limits the efficacy of some drugs, whereas peptide-based drugs offer advantages due to their ability to penetrate the BBB [141]. However, current studies lack quantitative data—such as brain-to-plasma concentration ratios and cerebrospinal fluid (CSF) penetration rates—making it impossible to accurately assess BBB penetration efficiency and effective intracerebral delivery levels accurately [23,104,105,125,129].

The oligopeptide C23, derived from the human CIRP protein (Ser111-Glu125), binds to the TLR4/MD2 receptor with exceptionally high affinity (Kd = 2.97 × 10^−8^ M) [9], and can competitively displace CIRP from binding to this complex. Its anti-inflammatory and anti-injury effects have been demonstrated in various multi-system disease models, including acute pancreatitis, sepsis, and organ ischemia–reperfusion injury [98,142,143,144,145,146,147,148,149,150,151,152,153,154,155,156,157]. Additionally, surface plasmon resonance (SPR) experiments have confirmed that C23 specifically blocks the binding of eCIRP to IL-6Rα [125]. At a concentration of 25 μM, C23 increases the equilibrium dissociation constant (Kd) of their binding from 8.08 × 10^−8^ M to 3.43 × 10^−6^ M, representing a 40-fold decrease in affinity; complete binding inhibition is achieved at 50 μM [125]. In neurological diseases, C23 targets the eCIRP-TLR4 pathway as a central mechanism. In the transient middle cerebral artery occlusion (tMCAO) mouse model of IS, administration of C23 at 8 μg/g body weight via retro-orbital injection attenuates the eCIRP-induced upregulation of miR-155, restores MerTK expression and phagocytic function in microglia, and exerts anti-inflammatory and neuroprotective effects [23]. Considering the characteristic of early increase and gradual decrease 3 days later of serum CIRP in IS patients [90], C23 administration should be initiated as early as possible and not exceed 3 days. Given the pathological involvement of the eCIRP-TLR4 pathway in IS, cerebral IR, ICH, TBI, and AD [21,22,23,24,25,26,27], C23 is expected to exert broad-spectrum protective effects in various neurological disorders. Furthermore, C23 has been shown to specifically block the binding of eCIRP to IL-6Rα, inhibit the IL-6Rα/STAT3/Cdk5 and IL-6Rα/PLC/IP3 signaling pathway, and have translational potential in eCIRP-related AD therapy [105,125]. However, C23 research has obvious limitations: a lack of quantitative data such as intracerebral accumulation concentration after BBB penetration, brain-to-plasma concentration ratios, and CSF penetration rate, making it impossible to confirm whether the high in vitro affinity (Kd = 2.97 × 10^−8^ M) can exert effects in vivo [9,23,105,125]; small sample size (3–8 animals per group), focusing on young male models, lacking verification in elderly, female, and models with comorbidities; single model, short intervention cycle, unclear long-term efficacy and multi-laboratory reproducibility; lack of pharmacokinetic data, limiting the design of clinical administration regimens; failure to clarify protective differences among different neuron subtypes, and absence of direct efficacy data in non-human primates, requiring further verification of translational potential [23,105,125].

Another type of peptide inhibitor, Tat-CIRP-CMA (TCC), penetrates the BBB through the Tat transmembrane domain (YGRKKRRQRRR), and its CIRP 106–125 domain can dual-target MD2 and IL-6Rα [104,129]. SPR experiments confirmed that the binding affinity of TCC to IL-6Rα reaches 4.12 × 10^−9^ M, and 1.5 μg/mL TCC can significantly reduce the binding affinity of eCIRP to IL-6Rα (Kd increased from 4.27 × 10^−9^ M to 1.77 × 10^−8^ M) [104]. In the in vitro oxygen-glucose deprivation (OGD) model pre-incubated with TCC for 1 h and the rat ICH model administered with TCC 2 h after hemorrhage, TCC exerts anti-inflammatory effects by reducing neuronal CIRP release, inhibiting the activation of the IL-6R/STAT3 pathway in microglia, enhancing microglial phagocytic function to decrease hematoma volume, and reducing the levels of inflammatory factors such as TNF-α and IL-6 [104], offering a novel candidate for precision-targeted ICH therapy. Given that eCIRP promotes tau phosphorylation via the IL-6Rα/STAT3/Cdk5 axis in AD patients [27], TCC theoretically holds potential to delay AD progression, although this hypothesis requires further experimental validation. In addition, in the perioperative neurocognitive disorder (PND) model with intravenous injection of TCC for 3 consecutive days after surgery, TCC can also improve anesthesia-surgery-induced cognitive impairment by degrading MD2 and downregulating the expression of membrane α5GABAARs and their tonic currents in hippocampal CA1 pyramidal neurons [129], indicating its multi-scenario application potential in neuroinflammation-related diseases. However, TCC research also has shortcomings: although its BBB penetration efficiency has been verified by Cy7 labeling, it lacks quantitative data such as actual intracerebral drug concentration, brain-to-plasma concentration ratio, and CSF penetration rate, making it impossible to determine whether the high in vitro affinity (4.12 × 10^−9^ M) can effectively block eCIRP binding in vivo; limited model coverage (lack of clinically relevant models such as hypertensive ICH), small sample size (6–10 animals per group) without multi-center verification; unknown long-term safety and intracerebral accumulation effects, with a maximum intervention cycle of only 28 days; outcome indicators do not cover long-term pathological processes such as cerebrovascular repair and neurogenesis, nor do they compare efficacy with commonly used clinical drugs [104,129].

The primary advantages of C23 and TCC lie in their strong target specificity and BBB penetration ability [23,104,129,151], but both face significant common problems. First, their pharmacokinetic properties remain unclear, and inherent issues associated with peptide drugs—such as short half-life and susceptibility to protease degradation—have yet to be resolved [141]. Second, there is a lack of quantitative data on drug delivery following BBB penetration, making it difficult to confirm whether sufficient drug concentrations reach intracerebral targets [23,104,105,125,129,141]. If intracerebral concentrations fail to reach the micromolar range, this may create a translational barrier between high in vitro activity and low in vivo efficacy. To date, neither of these two drugs has entered the clinical trial stage, lacking evidence for CNS-related clinical application. From a clinical translation perspective, existing studies have demonstrated that TCC can distribute within the brain via Cy7 labeling; however, quantitative data such as intracerebral concentration, brain-to-plasma concentration ratio, and CSF penetration rate have not been provided. This absence hinders accurate assessment of its BBB penetration efficiency and effective intracerebral delivery [104,129]. In contrast, C23 has not undergone any quantitative evaluation related to BBB penetration, with its ability inferred only indirectly through in vivo efficacy studies [23,105,125]. Therefore, C23 requires clarification of BBB penetration-related quantitative parameters using techniques such as microdialysis to verify correlations among in vitro affinity, intracerebral concentration, and in vivo efficacy. Similarly, TCC should quantify these parameters and establish a dose–response relationship curve. Furthermore, the inhibitory effects of C23 on the IL-6Rα/STAT3/Cdk5 and IL-6Rα/PLC/IP3 pathways need further validation in in vivo disease models, such as AD, to elucidate its long-term impact on tau phosphorylation and neurofibrillary tangle formation [105,125]. Future research should also be enhanced by expanding sample sizes, covering more clinically relevant models (e.g., elderly, female subjects, and those with comorbidities), supplementing long-term safety and pharmacokinetic data, and conducting multi-center reproducibility studies. At the same time, systemic inhibition of eCIRP may have potential off-target effects, such as interfering with normal immune regulation and affecting peripheral tissue function. Strategies such as targeted drug delivery to reduce peripheral drug concentration, short-term administration for acute diseases, and minimum effective dose for chronic diseases can be considered to avoid risks. Targeted drug delivery can rely on technologies such as focused ultrasound to transiently open the BBB, coupling with BBB shuttle peptides targeting LRP-1 receptors such as Angiopep-2, or using PLGA nanocarriers to encapsulate peptides and modify transferrin or lactoferrin ligands on the surface to protect peptides from degradation and enhance intracerebral targeted enrichment, and provide technical support for quantitative delivery after BBB penetration, laying the foundation for the clinical translation of such drugs [27].

In addition, CIRP function is closely related to its nucleocytoplasmic localization. Blocking its nucleocytoplasmic translocation can dual-regulate inflammatory responses and gene expression networks, providing a key target for intervention. However, there are currently no specific small-molecule drugs targeting this process, which is an important direction for subsequent research [3,6].

It is noteworthy that significant knowledge gaps persist in current CIRP research on neurological disorders. Key biological variables, such as sex and age, have not been systematically investigated for their impact on CIRP function. Neurological diseases like IS, AD, and TBI exhibit pronounced differences related to sex and age. For example, the incidence of AD is higher in women than in men [158], and elderly stroke patients experience poorer outcomes with distinct inflammatory responses compared to younger patients [159]. However, existing studies predominantly utilize young male animal models and often lack female control groups or aged models for comparative analysis. This limitation hinders clarification of whether CIRP expression levels, the severity of pathological impact, and responses to therapeutic interventions vary across different sexes and age groups. Clinical studies also lack stratified analyses by sex and age, making it difficult to determine whether eCIRP biomarker thresholds require sex-specific adjustments, thereby limiting its clinical translational applicability [90,104]. Furthermore, the mechanisms by which sex hormones (e.g., estrogen, androgens) or age-related signaling pathways (e.g., SIRT1, mTOR) regulate CIRP transcription, nuclear–cytoplasmic transport, and extracellular release remain entirely unknown.

Future research should focus on the biphasic nature of CIRP—its intracellular protective role and extracellular damaging effects—addressing gaps in core mechanisms and translational challenges, and constructing a complete translational chain. First, employ CRISPR-Cas9 gene editing, live-cell imaging, and other technologies to elucidate the molecular switches regulating nuclear–cytoplasmic transport, and utilize cross-disease model experiments to clarify the synergistic interactions between the TLR4 pathway and disease-specific pathways. Second, conduct multicenter, large-cohort studies to standardize the timing and threshold values for detecting eCIRP as a biomarker in diseases such as IS, ICH, and AD, establish a diagnostic model by combining multiple biomarkers, and innovate detection technologies to solve clinical application barriers. Third, optimize intracerebral delivery efficiency and pharmacokinetic profiles of peptide therapeutics like C23 and TCC, supplement long-term safety data, and promote clinical trial translation. Fourth, incorporate biological variables such as gender and age, conduct stratified studies to clarify the functional differences and regulatory mechanisms of CIRP in different populations, thereby laying the groundwork for personalized treatment. Fifth, explore combined treatment strategies integrating hypothermia with targeted therapeutics. By leveraging multi-omics and targeted imaging technologies to monitor treatment effects dynamically, ultimately advance CIRP research from mechanistic studies toward precise clinical translation, providing innovative diagnostic and therapeutic solutions for neurological disorders.

## Figures and Tables

**Table 1:** Summary of microenvironment, eCIRP cellular sources, and related mechanisms (commonalities and specificities) in neurological disorders.

Disease Type	Disease Microenvironment Characteristics	eCIRP Cellular Sources	Common Mechanisms	Disease-Specific Mechanisms	References
IS	Acute ischemia-hypoxia, vascular endothelial injury, inflammatory cell infiltration	Neurons, Microglia	1. eCIRP release; 2. Activation of TLR4-mediated neuroinflammation; 3. Neuronal injury/apoptosis	1. Induces neutrophil NET formation via the TLR4/p38 pathway and disrupts the BBB; 2. Induces microglial miR-155 expression, inhibits the MafB/MerTK axis, and impairs phagocytic function	[21,22,23,102,112]
Cerebral I/R injury	Post-ischemic reperfusion, oxidative stress outburst, mitochondrial dysfunction	Neurons, Microglia, Astrocytes		Promotes neuroinflammation via the TLR4/NF-κB/NLRP3 inflammasome pathway	[24,119]
ICH	Hematoma microenvironment, heme accumulation, local compressive injury	Neurons, Microglia		Activates the IL-6Rα/STAT3 signaling pathway to drive microglial pro-inflammatory polarization	[26,104]
TBI	Mechanical tissue damage, acute cell rupture, stress pathway activation	Neurons, Microglia		1. Activates microglia via the TLR4-mediated Histone H3/α7nAChR signaling axis; 2. Activates the ERS-related PERK/ATF4/CHOP pathway to promote the Caspase-3-dependent apoptotic pathway	[25]
AD	Aβ plaque deposition, tau tangles, chronic inflammatory microenvironment	Microglia		Regulates tau/Aβ metabolic disorders: 1. Activate the IL-6Rα/STAT3/Cdk5 signaling pathway; 2. Activate the IL-6Rα/PLC/IP_3_ pathway; 3. CIRP overexpression in astrocytes promotes Aβ1–42 production; 4. Regulates HMGB1 release	[27,94,105,107,125,126]

## Data Availability

Data availability is not applicable to this article as no new data were created or analyzed in this study.

## Abbreviations

The following abbreviations are used in this manuscript:

3′ UTR	3′ untranslated region
AD	Alzheimer’s disease
Akt	Protein kinase B
ALT	Alanine aminotransferase
AQP4	Aquaporin 4
AST	Aspartate aminotransferase
ATF4	Activating transcription factor 4
Aβ	β-amyloid
Bax	B lymphoblastoma-2 gene-associated X protein
BBB	Blood–brain barrier
Bcl-2	B lymphoblastoma-2 gene
BRD2	Bromodomain-containing protein 2
BWC	Brain water content
Caspase-1	Cysteine aspartic protease-1
Caspase-3	Cysteine aspartic protease-3
Cdk5	Cytokinin-dependent protein kinase 5
CHOP	DNA damage-inducible transcript 3
CIRP	Cold-inducible RNA-binding protein
CIRBP	Cold-inducible RNA-binding protein
CNS	Central nervous system
CPB	Cardiopulmonary bypass
CRD	Circadian rhythm disruption
CRP	C-reactive protein
CSF	cerebrospinal fluid
DAMP	Damage-associated molecular pattern
DLB	Dementia with Lewy bodies
eCIRP	Extracellular cold-inducible RNA-binding protein
EGF	Epidermal growth factor
ER	Endoplasmic reticulum
ERK	Extracellular signal-regulated kinase
ERS	Endoplasmic reticulum stress
FTD	Frontotemporal dementia
GSDMD	Gasdermin D
GSK3β	Glycogen synthase kinase 3β
H_2_O_2_	Hydrogen peroxide
HD	Huntington’s disease
HIF-1α	Hypoxia-inducible factor-1α
HMGB1	High mobility group protein B1
hnRNP A18	Heterogeneous nuclear ribonucleoprotein A18
I/R	Ischemia–reperfusion
ICH	Intracerebral hemorrhage
iCIRP	Intracellular cold-inducible RNA-binding protein
IFN-β	Interferon-β
IL-13	Interleukin 13
IL-1β	Interleukin 1β
IL-5	Interleukin 5
IL-6	Interleukin 6
IL-6R	Interleukin 6 receptor
IL-6Rα	Interleukin-6 receptor α
IP3	Inositol 1,4,5-trisphosphate
IP3R	Inositol 1,4,5-trisphosphate receptor
ipRGCs	Intrinsically photosensitive retinal ganglion cells
IRAK	Interleukin-1 receptor-associated kinase
IRF3	Interferon regulatory factor 3
IS	Ischemic stroke
ISWRD	Irregular sleep–wake rhythm disorder
JNK	c-Jun N-terminal kinase
LDH	Lactate dehydrogenase
MafB	MAF bZIP transcription factor B
MAM	Mitochondria-associated endoplasmic reticulum membrane
MAPK	Mitogen-activated protein kinase
MD2	Myeloid differentiation protein-2
MDA	Malondialdehyde
MerTK	MER proto-oncogene, tyrosine kinase
miR-155	MicroRNA-155
MMP-9	Matrix metalloproteinase-9
MSA	Multiple system atrophy
MyD88	Myeloid differentiation factor 88
NCOR1	Nuclear receptor corepressor 1
NDS	Neurological deficit scores
NET	Neutrophil extracellular trap
NF-κB	Nuclear factor kappa-B
NIH3T3	National Institutes of Health 3-day transfer, inoculum 3 × 10^5^ cells
NIHSS	National Institute of Health stroke scale
NLRP3	NOD-like receptor thermal protein domain associated protein 3
OGD	Oxygen-glucose deprivation
p38	p38 mitogen-activated protein kinase
PD	Parkinson’s disease
Per	Period circadian regulator
PER3	Period circadian regulator 3
PERK	Protein kinase R-like endoplasmic reticulum kinase
PLC	Phospholipase C
PND	Perioperative neurocognitive disorder
RBD	Rapid eye movement sleep behavior disorder
RHT	Retinal-hypothalamic tract
RORα	Retinoic acid receptor-related orphan receptor-α
ROS	Reactive oxygen species
RRM	RNA recognition motif
SAH	Subarachnoid hemorrhage
SCN	Suprachiasmatic nucleus
SIRT1	Silent information regulator 1
Sp1	Transcription factor sp1
STAT3	Signal transducer and activator of transcription 3
TBI	Traumatic brain injury
TBK1	TANK binding kinase 1
TCC	Tat-CIRP-CMA
TGF-β1	Transforming growth factor-β1
TLR4	Toll-like receptor 4
tMCAO	Transient middle cerebral artery occlusion
TNF-α	Tumor necrosis factor-α
TRAF3	TNF receptor associated factor 3
TREM-1	Triggering receptor expressed on myeloid cells-1
TRIF	TIR-domain- containing adapter-inducing interferon-β
TRX	Thioredoxin
uPA	Urokinase-type plasminogen activator
VDAC1	Voltage-dependent anion channel 1
α7nAChR	Nicotinic acetylcholine receptor α7 subunit

## References

[1] Nishiyama H., Higashitsuji H., Yokoi H., Itoh K., Danno S., Matsuda T., Fujita J. (1997). Cloning and characterization of human CIRP (cold-inducible RNA-binding protein) cDNA and chromosomal assignment of the gene. Gene.

[2] Nishiyama H., Itoh K., Kaneko Y., Kishishita M., Yoshida O., Fujita J. (1997). A glycine-rich RNA-binding protein mediating cold-inducible suppression of mammalian cell growth. J. Cell Biol..

[3] Zhong P., Huang H. (2017). Recent progress in the research of cold-inducible RNA-binding protein. Future Sci. OA.

[4] Zhong P., Peng J., Bian Z., Huang H. (2021). The Role of Cold Inducible RNA-Binding Protein in Cardiac Physiology and Diseases. Front. Pharmacol..

[5] Chen M., Fu H., Zhang J., Huang H., Zhong P. (2019). CIRP downregulation renders cardiac cells prone to apoptosis in heart failure. Biochem. Biophys. Res. Commun..

[6] Aziz M., Brenner M., Wang P. (2019). Extracellular CIRP (eCIRP) and inflammation. J. Leukoc. Biol..

[7] Zhong P., Peng J., Yuan M., Kong B., Huang H. (2021). Cold-inducible RNA-binding protein (CIRP) in inflammatory diseases: Molecular insights of its associated signalling pathways. Scand. J. Immunol..

[8] Zhong P., Zhou M., Zhang J., Peng J., Zeng G., Huang H. (2022). The role of Cold-Inducible RNA-binding protein in respiratory diseases. J. Cell. Mol. Med..

[9] Qiang X., Yang W.L., Wu R., Zhou M., Jacob A., Dong W., Kuncewitch M., Ji Y., Yang H., Wang H. (2013). Cold-inducible RNA-binding protein (CIRP) triggers inflammatory responses in hemorrhagic shock and sepsis. Nat. Med..

[10] Bolognese A.C., Sharma A., Yang W.L., Nicastro J., Coppa G.F., Wang P. (2018). Cold-inducible RNA-binding protein activates splenic T cells during sepsis in a TLR4-dependent manner. Cell. Mol. Immunol..

[11] Liao Y., Feng J., Sun W., Wu C., Li J., Jing T., Liang Y., Qian Y., Liu W., Wang H. (2021). CIRP promotes the progression of non-small cell lung cancer through activation of Wnt/β-catenin signaling via CTNNB1. J. Exp. Clin. Cancer Res. CR.

[12] Han J., Zhang Y., Ge P., Dakal T.C., Wen H., Tang S., Luo Y., Yang Q., Hua B., Zhang G. (2023). Exosome-derived CIRP: An amplifier of inflammatory diseases. Front. Immunol..

[13] Godwin A., Yang W.L., Sharma A., Khader A., Wang Z., Zhang F., Nicastro J., Coppa G.F., Wang P. (2015). Blocking cold-inducible RNA-binding protein protects liver from ischemia-reperfusion injury. Shock.

[14] Liu M., Li Y., Liu Y., Yan S., Liu G., Zhang Q., Ji B. (2019). Cold-inducible RNA-binding protein as a novel target to alleviate blood-brain barrier damage induced by cardiopulmonary bypass. J. Thorac. Cardiovasc. Surg..

[15] Saito K., Fukuda N., Matsumoto T., Iribe Y., Tsunemi A., Kazama T., Yoshida-Noro C., Hayashi N. (2010). Moderate low temperature preserves the stemness of neural stem cells and suppresses apoptosis of the cells via activation of the cold-inducible RNA binding protein. Brain Res..

[16] Chen X., Liu X., Li B., Zhang Q., Wang J., Zhang W., Luo W., Chen J. (2017). Cold Inducible RNA Binding Protein Is Involved in Chronic Hypoxia Induced Neuron Apoptosis by Down-Regulating HIF-1α Expression and Regulated By microRNA-23a. Int. J. Biol. Sci..

[17] Chen L., Tian Q., Wang W. (2019). Association between CIRP expression and hypoxic-ischemic brain injury in neonatal rats. Exp. Ther. Med..

[18] Sakurai T., Itoh K., Higashitsuji H., Nonoguchi K., Liu Y., Watanabe H., Nakano T., Fukumoto M., Chiba T., Fujita J. (2006). Cirp protects against tumor necrosis factor-alpha-induced apoptosis via activation of extracellular signal-regulated kinase. Biochim. et Biophys. Acta.

[19] Wang G., Zhang J.N., Guo J.K., Cai Y., Sun H.S., Dong K., Wu C.G. (2016). Neuroprotective effects of cold-inducible RNA-binding protein during mild hypothermia on traumatic brain injury. Neural Regen. Res..

[20] Liu J., Xue J., Zhang H., Li S., Liu Y., Xu D., Zou M., Zhang Z., Diao J. (2015). Cloning, expression, and purification of cold inducible RNA-binding protein and its neuroprotective mechanism of action. Brain Res..

[21] Li Z., Sun S., Xiao Q., Tan S., Jin H., Hu B. (2024). Neuron Derived Cold-Inducible RNA-Binding Protein Promotes NETs Formation to Exacerbate Brain Endothelial Barrier Disruption after Ischemic Stroke. Aging Dis..

[22] Zhou M., Yang W.L., Ji Y., Qiang X., Wang P. (2014). Cold-inducible RNA-binding protein mediates neuroinflammation in cerebral ischemia. Biochim. et Biophys. Acta.

[23] Lapin D., Aylar D., Sharma A., Wang P. (2025). Extracellular CIRP dysregulates microglial efferocytosis in ischemic stroke via the TLR4/miR-155/MafB axis. Res. Sq..

[24] Fan Y., Wei J., Lin L., Lin J., Li X., Xu L., Zhou X., Li Y., Yang Y. (2025). Cold-Induced RNA-Binding Protein (CIRP) Affects Cerebral Ischemia-Reperfusion Injury Through NF-κB Pathway. Mol. Neurobiol..

[25] Liu Y.X., Zhao M., Yu Y., Liu J.P., Liu W.J., Yao R.Q., Wang J., Yang R.L., Wu Y., Dong N. (2024). Extracellular cold-inducible RNA-binding protein mediated neuroinflammation and neuronal apoptosis after traumatic brain injury. Burn. Trauma.

[26] Zhou K., Cui S., Duan W., Zhang J., Huang J., Wang L., Gong Z., Zhou Y. (2020). Cold-inducible RNA-binding protein contributes to intracerebral hemorrhage-induced brain injury via TLR4 signaling. Brain Behav..

[27] Sharma A., Brenner M., Wang P. (2020). Potential Role of Extracellular CIRP in Alcohol-Induced Alzheimer’s Disease. Mol. Neurobiol..

[28] Saito T., Sugimoto K., Adachi Y., Wu Q., Mori K.J. (2000). Cloning and characterization of amphibian cold inducible RNA-binding protein. Comp. Biochem. Physiol. Part B Biochem. Mol. Biol..

[29] Nishiyama H., Xue J.H., Sato T., Fukuyama H., Mizuno N., Houtani T., Sugimoto T., Fujita J. (1998). Diurnal change of the cold-inducible RNA-binding protein (Cirp) expression in mouse brain. Biochem. Biophys. Res. Commun..

[30] Nikonova E.V., Gilliland J.D., Tanis K.Q., Podtelezhnikov A.A., Rigby A.M., Galante R.J., Finney E.M., Stone D.J., Renger J.J., Pack A.I. (2017). Transcriptional Profiling of Cholinergic Neurons From Basal Forebrain Identifies Changes in Expression of Genes Between Sleep and Wake. Sleep.

[31] Morf J., Rey G., Schneider K., Stratmann M., Fujita J., Naef F., Schibler U. (2012). Cold-inducible RNA-binding protein modulates circadian gene expression posttranscriptionally. Science.

[32] Sugimoto K., Jiang H. (2008). Cold stress and light signals induce the expression of cold-inducible RNA binding protein (cirp) in the brain and eye of the Japanese treefrog (Hyla japonica). Comp. Biochem. Physiol. Part A Mol. Integr. Physiol..

[33] Güler A.D., Ecker J.L., Lall G.S., Haq S., Altimus C.M., Liao H.W., Barnard A.R., Cahill H., Badea T.C., Zhao H. (2008). Melanopsin cells are the principal conduits for rod-cone input to non-image-forming vision. Nature.

[34] Hannibal J., Hindersson P., Knudsen S.M., Georg B., Fahrenkrug J. (2002). The photopigment melanopsin is exclusively present in pituitary adenylate cyclase-activating polypeptide-containing retinal ganglion cells of the retinohypothalamic tract. J. Neurosci..

[35] Hattar S., Liao H.W., Takao M., Berson D.M., Yau K.W. (2002). Melanopsin-containing retinal ganglion cells: Architecture, projections, and intrinsic photosensitivity. Science.

[36] Riedel C.S., Georg B., Hannibal J. (2023). Phenotyping of light-activated neurons in the mouse SCN based on the expression of FOS and EGR1. Front. Physiol..

[37] Leng Y., Musiek E.S., Hu K., Cappuccio F.P., Yaffe K. (2019). Association between circadian rhythms and neurodegenerative diseases. Lancet Neurol..

[38] Nassan M., Videnovic A. (2022). Circadian rhythms in neurodegenerative disorders. Nat. Rev. Neurol..

[39] Volicer L., Harper D.G., Manning B.C., Goldstein R., Satlin A. (2001). Sundowning and circadian rhythms in Alzheimer’s disease. Am. J. Psychiatry.

[40] Canevelli M., Valletta M., Trebbastoni A., Sarli G., D’Antonio F., Tariciotti L., de Lena C., Bruno G. (2016). Sundowning in Dementia: Clinical Relevance, Pathophysiological Determinants, and Therapeutic Approaches. Front. Med..

[41] Auger R.R., Burgess H.J., Emens J.S., Deriy L.V., Thomas S.M., Sharkey K.M. (2015). Clinical Practice Guideline for the Treatment of Intrinsic Circadian Rhythm Sleep-Wake Disorders: Advanced Sleep-Wake Phase Disorder (ASWPD), Delayed Sleep-Wake Phase Disorder (DSWPD), Non-24-Hour Sleep-Wake Rhythm Disorder (N24SWD), and Irregular Sleep-Wake Rhythm Disorder (ISWRD). An Update for 2015: An American Academy of Sleep Medicine Clinical Practice Guideline. J. Clin. Sleep Med..

[42] Abbott S.M., Zee P.C. (2015). Irregular Sleep-Wake Rhythm Disorder. Sleep Med. Clin..

[43] Madamanchi K., Zhang J., Melkani G.C. (2025). Linkage of circadian rhythm disruptions with Alzheimer’s disease and therapeutic interventions. Acta Pharm. Sin. B.

[44] Videnovic A., Noble C., Reid K.J., Peng J., Turek F.W., Marconi A., Rademaker A.W., Simuni T., Zadikoff C., Zee P.C. (2014). Circadian melatonin rhythm and excessive daytime sleepiness in Parkinson disease. JAMA Neurol..

[45] Tholfsen L.K., Larsen J.P., Schulz J., Tysnes O.B., Gjerstad M.D. (2015). Development of excessive daytime sleepiness in early Parkinson disease. Neurology.

[46] Breen D.P., Vuono R., Nawarathna U., Fisher K., Shneerson J.M., Reddy A.B., Barker R.A. (2014). Sleep and circadian rhythm regulation in early Parkinson disease. JAMA Neurol..

[47] Zhong G., Bolitho S., Grunstein R., Naismith S.L., Lewis S.J. (2013). The relationship between thermoregulation and REM sleep behaviour disorder in Parkinson’s disease. PLoS ONE.

[48] Berganzo K., Díez-Arrola B., Tijero B., Somme J., Lezcano E., Llorens V., Ugarriza I., Ciordia R., Gómez-Esteban J.C., Zarranz J.J. (2013). Nocturnal hypertension and dysautonomia in patients with Parkinson’s disease: Are they related?. J. Neurol..

[49] Bolitho S.J., Naismith S.L., Rajaratnam S.M., Grunstein R.R., Hodges J.R., Terpening Z., Rogers N., Lewis S.J. (2014). Disturbances in melatonin secretion and circadian sleep-wake regulation in Parkinson disease. Sleep Med..

[50] Chahine L.M., Amara A.W., Videnovic A. (2017). A systematic review of the literature on disorders of sleep and wakefulness in Parkinson’s disease from 2005 to 2015. Sleep Med. Rev..

[51] De Pablo-Fernández E., Courtney R., Warner T.T., Holton J.L. (2018). A Histologic Study of the Circadian System in Parkinson Disease, Multiple System Atrophy, and Progressive Supranuclear Palsy. JAMA Neurol..

[52] Raupach A.K., Ehgoetz Martens K.A., Memarian N., Zhong G., Matar E., Halliday G.M., Grunstein R., Lewis S.J.G. (2020). Assessing the role of nocturnal core body temperature dysregulation as a biomarker of neurodegeneration. J. Sleep Res..

[53] McKeith I.G., Boeve B.F., Dickson D.W., Halliday G., Taylor J.P., Weintraub D., Aarsland D., Galvin J., Attems J., Ballard C.G. (2017). Diagnosis and management of dementia with Lewy bodies: Fourth consensus report of the DLB Consortium. Neurology.

[54] Harper D.G., Stopa E.G., McKee A.C., Satlin A., Harlan P.C., Goldstein R., Volicer L. (2001). Differential circadian rhythm disturbances in men with Alzheimer disease and frontotemporal degeneration. Arch. Gen. Psychiatry.

[55] Musiek E.S., Lim M.M., Yang G., Bauer A.Q., Qi L., Lee Y., Roh J.H., Ortiz-Gonzalez X., Dearborn J.T., Culver J.P. (2013). Circadian clock proteins regulate neuronal redox homeostasis and neurodegeneration. J. Clin. Investig..

[56] Amidfar M., Garcez M.L., Kim Y.K. (2023). The shared molecular mechanisms underlying aging of the brain, major depressive disorder, and Alzheimer’s disease: The role of circadian rhythm disturbances. Prog. Neuro-Psychopharmacol. Biol. Psychiatry.

[57] Chen X., Kondo K., Motoki K., Homma H., Okazawa H. (2015). Fasting activates macroautophagy in neurons of Alzheimer’s disease mouse model but is insufficient to degrade amyloid-beta. Sci. Rep..

[58] He Y., Cornelissen-Guillaume G.G., He J., Kastin A.J., Harrison L.M., Pan W. (2016). Circadian rhythm of autophagy proteins in hippocampus is blunted by sleep fragmentation. Chronobiol. Int..

[59] McKee C.A., Lananna B.V., Musiek E.S. (2020). Circadian regulation of astrocyte function: Implications for Alzheimer’s disease. Cell. Mol. Life Sci. CMLS.

[60] Idris Z., Zenian M.S., Muzaimi M., Hamid W.Z. (2014). Better Glasgow outcome score, cerebral perfusion pressure and focal brain oxygenation in severely traumatized brain following direct regional brain hypothermia therapy: A prospective randomized study. Asian J. Neurosurg..

[61] Zhang H.T., Xue J.H., Zhang Z.W., Kong H.B., Liu A.J., Li S.C., Xu D.G. (2015). Cold-inducible RNA-binding protein inhibits neuron apoptosis through the suppression of mitochondrial apoptosis. Brain Res..

[62] Gao Y., Liu H., Zhou Y., Cai S., Zhang J., Sun J., Duan M. (2024). Cold inducible RNA binding protein-regulated mitochondria associated endoplasmic reticulum membranes-mediated Ca(2+) transport play a critical role in hypothermia cerebral resuscitation. Exp. Neurol..

[63] Wu L., Sun H.L., Gao Y., Hui K.L., Xu M.M., Zhong H., Duan M.L. (2017). Therapeutic Hypothermia Enhances Cold-Inducible RNA-Binding Protein Expression and Inhibits Mitochondrial Apoptosis in a Rat Model of Cardiac Arrest. Mol. Neurobiol..

[64] Li J.H., Zhang X., Meng Y., Li C.S., Ji H., Yang H.M., Li S.Z. (2015). [Cold inducible RNA-binding protein inhibits hippocampal neuronal apoptosis under hypothermia by regulating redox system]. Sheng Li Xue Bao [Acta Physiol. Sin.].

[65] Li S., Zhang Z., Xue J., Liu A., Zhang H. (2012). Cold-inducible RNA binding protein inhibits H_2_O_2_-induced apoptosis in rat cortical neurons. Brain Res..

[66] Yang R., Weber D.J., Carrier F. (2006). Post-transcriptional regulation of thioredoxin by the stress inducible heterogenous ribonucleoprotein A18. Nucleic Acids Res..

[67] Lu W., Jiang Z., Wen J. (2025). The pathological roles of thioredoxin-interacting protein in ischemic stroke, focusing on oxidative stress and pyroptosis. Int. J. Biol. Macromol..

[68] Salameh A., Dhein S., Dähnert I., Klein N. (2016). Neuroprotective Strategies during Cardiac Surgery with Cardiopulmonary Bypass. Int. J. Mol. Sci..

[69] Vedel A.G., Holmgaard F., Danielsen E.R., Langkilde A., Paulson O.B., Ravn H.B., Rasmussen L.S., Nilsson J.C. (2020). Blood pressure and brain injury in cardiac surgery: A secondary analysis of a randomized trial. Eur. J. Cardio-Thorac. Surg..

[70] Wan Z., Li Y., Ye H., Zi Y., Zhang G., Wang X. (2021). Plasma S100β and neuron-specific enolase, but not neuroglobin, are associated with early cognitive dysfunction after total arch replacement surgery: A pilot study. Medicine.

[71] Liu A., Zhang Z., Li A., Xue J. (2010). Effects of hypothermia and cerebral ischemia on cold-inducible RNA-binding protein mRNA expression in rat brain. Brain Res..

[72] Sanderson T.H., Reynolds C.A., Kumar R., Przyklenk K., Hüttemann M. (2013). Molecular mechanisms of ischemia-reperfusion injury in brain: Pivotal role of the mitochondrial membrane potential in reactive oxygen species generation. Mol. Neurobiol..

[73] Sies H., Jones D.P. (2020). Reactive oxygen species (ROS) as pleiotropic physiological signalling agents. Nat. Rev. Mol. Cell Biol..

[74] Cheng J., Wang F., Yu D.F., Wu P.F., Chen J.G. (2011). The cytotoxic mechanism of malondialdehyde and protective effect of carnosine via protein cross-linking/mitochondrial dysfunction/reactive oxygen species/MAPK pathway in neurons. Eur. J. Pharmacol..

[75] Cai J., Chen J., He H., Yin Z., Zhu Z., Yin D. (2009). Carbonyl stress: Malondialdehyde induces damage on rat hippocampal neurons by disturbance of Ca(2+) homeostasis. Cell Biol. Toxicol..

[76] Chen J., Bie Y., Guan Y., Liu W., Xu F., Liu T., Meng Z., Gao M., Liu J., Xie S. (2024). Ischemic Stroke Induces ROS Accumulation, Maladaptive Mitophagy, and Neuronal Apoptosis in Minipigs. J. Microbiol. Biotechnol..

[77] Xue J.H., Nonoguchi K., Fukumoto M., Sato T., Nishiyama H., Higashitsuji H., Itoh K., Fujita J. (1999). Effects of ischemia and H2O2 on the cold stress protein CIRP expression in rat neuronal cells. Free Radic. Biol. Med..

[78] McGinn J.T., Aziz M., Zhang F., Yang W.L., Nicastro J.M., Coppa G.F., Wang P. (2018). Cold-inducible RNA-binding protein-derived peptide C23 attenuates inflammation and tissue injury in a murine model of intestinal ischemia-reperfusion. Surgery.

[79] Cen C., Yang W.L., Yen H.T., Nicastro J.M., Coppa G.F., Wang P. (2016). Deficiency of cold-inducible ribonucleic acid-binding protein reduces renal injury after ischemia-reperfusion. Surgery.

[80] Dai H., Zhou Y., Lu Y., Zhang X., Zhuang Z., Gao Y., Liu G., Chen C., Ma J., Li W. (2022). Decreased Expression of CIRP Induced by Therapeutic Hypothermia Correlates with Reduced Early Brain Injury after Subarachnoid Hemorrhage. J. Clin. Med..

[81] Schallner N., Pandit R., LeBlanc R., Thomas A.J., Ogilvy C.S., Zuckerbraun B.S., Gallo D., Otterbein L.E., Hanafy K.A. (2015). Microglia regulate blood clearance in subarachnoid hemorrhage by heme oxygenase-1. J. Clin. Investig..

[82] Garland P., Durnford A.J., Okemefuna A.I., Dunbar J., Nicoll J.A., Galea J., Boche D., Bulters D.O., Galea I. (2016). Heme-Hemopexin Scavenging Is Active in the Brain and Associates With Outcome After Subarachnoid Hemorrhage. Stroke.

[83] Yang Y., Chen S., Zhang J.M. (2017). The Updated Role of Oxidative Stress in Subarachnoid Hemorrhage. Curr. Drug Deliv..

[84] Zhang Z., Zhang A., Liu Y., Hu X., Fang Y., Wang X., Luo Y., Lenahan C., Chen S. (2022). New Mechanisms and Targets of Subarachnoid Hemorrhage: A Focus on Mitochondria. Curr. Neuropharmacol..

[85] Busija D.W., Leffler C.W. (1987). Hypothermia reduces cerebral metabolic rate and cerebral blood flow in newborn pigs. Am. J. Physiol..

[86] Lanier W.L. (1995). Cerebral metabolic rate and hypothermia: Their relationship with ischemic neurologic injury. J. Neurosurg. Anesthesiol..

[87] Zhou T., Mo J., Xu W., Hu Q., Liu H., Fu Y., Jiang J. (2023). Mild hypothermia alleviates oxygen-glucose deprivation/reperfusion-induced apoptosis by inhibiting ROS generation, improving mitochondrial dysfunction and regulating DNA damage repair pathway in PC12 cells. Apoptosis Int. J. Program. Cell Death.

[88] Xu J., Tao L., Jiang L., Lai J., Hu J., Tang Z. (2024). Moderate Hypothermia Alleviates Sepsis-Associated Acute Lung Injury by Suppressing Ferroptosis Induced by Excessive Inflammation and Oxidative Stress via the Keap1/GSK3β/Nrf2/GPX4 Signaling Pathway. J. Inflamm. Res..

[89] Liu M., Li Y., Gao S., Yan S., Zhang Q., Liu G., Ji B. (2020). A novel target to reduce microglial inflammation and neuronal damage after deep hypothermic circulatory arrest. J. Thorac. Cardiovasc. Surg..

[90] Li M., Yao M., Shao K., Shen X., Ge Z., Li Y. (2023). Serum cold-inducible RNA-binding protein (CIRP) levels as a prognostic indicator in patients with acute ischemic stroke. Front. Neurol..

[91] Li Z., Liu J.P., Yao F.H., Cao Y., Li S.C., Liu Y.Y., Wen S.X., Liu Y.X., Liu A.J. (2024). Cold Inducible RNA-Binding Protein Promotes the Development of Alzheimer’s Disease Partly by Inhibition of uPA in Astrocytes. Degener. Neurol. Neuromuscul. Dis..

[92] Sheth K.N. (2022). Spontaneous Intracerebral Hemorrhage. N. Engl. J. Med..

[93] Maas A.I.R., Menon D.K., Manley G.T., Abrams M., Åkerlund C., Andelic N., Aries M., Bashford T., Bell M.J., Bodien Y.G. (2022). Traumatic brain injury: Progress and challenges in prevention, clinical care, and research. Lancet Neurol..

[94] Scheltens P., De Strooper B., Kivipelto M., Holstege H., Chételat G., Teunissen C.E., Cummings J., van der Flier W.M. (2021). Alzheimer’s disease. Lancet.

[95] Jacob A., Ma Y., Nasiri E., Ochani M., Carrion J., Peng S., Brenner M., Huerta P.T., Wang P. (2019). Extracellular cold inducible RNA-binding protein mediates binge alcohol-induced brain hypoactivity and impaired cognition in mice. Mol. Med..

[96] Rehm J., Hasan O.S.M., Black S.E., Shield K.D., Schwarzinger M. (2019). Alcohol use and dementia: A systematic scoping review. Alzheimer’s Res. Ther..

[97] Ridley N.J., Draper B., Withall A. (2013). Alcohol-related dementia: An update of the evidence. Alzheimer’s Res. Ther..

[98] Denning N.L., Yang W.L., Hansen L., Prince J., Wang P. (2019). C23, an oligopeptide derived from cold-inducible RNA-binding protein, suppresses inflammation and reduces lung injury in neonatal sepsis. J. Pediatr. Surg..

[99] Kim H.J., Kim H., Lee J.H., Hwangbo C. (2023). Toll-like receptor 4 (TLR4): New insight immune and aging. Immun. Ageing.

[100] Wei X., Zhang F., Cheng D., Wang Z., Xing N., Yuan J., Zhang W., Xing F. (2024). Free heme induces neuroinflammation and cognitive impairment by microglial activation via the TLR4/MyD88/NF-κB signaling pathway. Cell Commun. Signal. CCS.

[101] Ali W., Choe K., Park J.S., Ahmad R., Park H.Y., Kang M.H., Park T.J., Kim M.O. (2024). Kojic acid reverses LPS-induced neuroinflammation and cognitive impairment by regulating the TLR4/NF-κB signaling pathway. Front. Pharmacol..

[102] Chen W., Wang L., Liu Z. (2021). MicroRNA-155 influences cell damage in ischemic stroke via TLR4/MYD88 signaling pathway. Bioengineered.

[103] Yamamoto M., Sato S., Hemmi H., Hoshino K., Kaisho T., Sanjo H., Takeuchi O., Sugiyama M., Okabe M., Takeda K. (2003). Role of adaptor TRIF in the MyD88-independent toll-like receptor signaling pathway. Science.

[104] Ye L., Tang X., Liu F., Wei T., Xu T., Jiang Z., Xu L., Xiang C., Yuan X., Shen L. (2025). Targeting CIRP and IL-6R-mediated microglial inflammation to improve outcomes in intracerebral hemorrhage. J. Adv. Res..

[105] Sharma A., Sari E., Lee Y., Patel S., Brenner M., Marambaud P., Wang P. (2023). Extracellular CIRP Induces Calpain Activation in Neurons via PLC-IP(3)-Dependent Calcium Pathway. Mol. Neurobiol..

[106] Laurent C., Buée L., Blum D. (2018). Tau and neuroinflammation: What impact for Alzheimer’s Disease and Tauopathies?. Biomed. J..

[107] Sarlus H., Heneka M.T. (2017). Microglia in Alzheimer’s disease. J. Clin. Investig..

[108] Lin S., Yin Q., Zhong Q., Lv F.L., Zhou Y., Li J.Q., Wang J.Z., Su B.Y., Yang Q.W. (2012). Heme activates TLR4-mediated inflammatory injury via MyD88/TRIF signaling pathway in intracerebral hemorrhage. J. Neuroinflamm..

[109] Campbell B.C.V., De Silva D.A., Macleod M.R., Coutts S.B., Schwamm L.H., Davis S.M., Donnan G.A. (2019). Ischaemic stroke. Nat. Rev. Dis. Primers.

[110] Abdullahi W., Tripathi D., Ronaldson P.T. (2018). Blood-brain barrier dysfunction in ischemic stroke: Targeting tight junctions and transporters for vascular protection. Am. J. Physiol. Cell Physiol..

[111] Ma Y., Yang X., Chatterjee V., Meegan J.E., Beard R.S., Yuan S.Y. (2019). Role of Neutrophil Extracellular Traps and Vesicles in Regulating Vascular Endothelial Permeability. Front. Immunol..

[112] Xie X.D., Dong S.S., Liu R.J., Shi L.L., Zhu T. (2024). Mechanism of Efferocytosis in Determining Ischaemic Stroke Resolution-Diving into Microglia/Macrophage Functions and Therapeutic Modality. Mol. Neurobiol..

[113] Molofsky A.V., Deneen B. (2015). Astrocyte development: A Guide for the Perplexed. Glia.

[114] Qin X., Wang J., Chen S., Liu G., Wu C., Lv Q., He X., Bai X., Huang W., Liao H. (2022). Astrocytic p75(NTR) expression provoked by ischemic stroke exacerbates the blood-brain barrier disruption. Glia.

[115] Liddelow S.A., Guttenplan K.A., Clarke L.E., Bennett F.C., Bohlen C.J., Schirmer L., Bennett M.L., Münch A.E., Chung W.S., Peterson T.C. (2017). Neurotoxic reactive astrocytes are induced by activated microglia. Nature.

[116] Hong S., Beja-Glasser V.F., Nfonoyim B.M., Frouin A., Li S., Ramakrishnan S., Merry K.M., Shi Q., Rosenthal A., Barres B.A. (2016). Complement and microglia mediate early synapse loss in Alzheimer mouse models. Science.

[117] Zhang Y., Gong X. (2024). Fat mass and obesity associated protein inhibits neuronal ferroptosis via the FYN/Drp1 axis and alleviate cerebral ischemia/reperfusion injury. CNS Neurosci. Ther..

[118] Li Y., Liao J., Xiong L., Xiao Z., Ye F., Wang Y., Chen T., Huang L., Chen M., Chen Z.S. (2024). Stepwise targeted strategies for improving neurological function by inhibiting oxidative stress levels and inflammation following ischemic stroke. J. Control. Release.

[119] Liu J., Ma W., Zang C.H., Wang G.D., Zhang S.J., Wu H.J., Zhu K.W., Xiang X.L., Li C.Y., Liu K.P. (2021). Salidroside inhibits NLRP3 inflammasome activation and apoptosis in microglia induced by cerebral ischemia/reperfusion injury by inhibiting the TLR4/NF-κB signaling pathway. Ann. Transl. Med..

[120] Chen W., Wu Z., Cheng Z., Zhang Y., Luo Q., Yin M. (2025). HO-1 represses NF-κB signaling pathway to mediate microglia polarization and phagocytosis in intracerebral hemorrhage. Neuroscience.

[121] Matloff W.J., Zhao L., Ning K., Conti D.V., Toga A.W. (2020). Interaction effect of alcohol consumption and Alzheimer disease polygenic risk score on the brain cortical thickness of cognitively normal subjects. Alcohol.

[122] Heymann D., Stern Y., Cosentino S., Tatarina-Nulman O., Dorrejo J.N., Gu Y. (2016). The Association Between Alcohol Use and the Progression of Alzheimer’s Disease. Curr. Alzheimer Res..

[123] Andrews S.J., Goate A., Anstey K.J. (2020). Association between alcohol consumption and Alzheimer’s disease: A Mendelian randomization study. Alzheimer’s Dement. J. Alzheimer’s Assoc..

[124] Schwarzinger M., Pollock B.G., Hasan O.S.M., Dufouil C., Rehm J. (2018). Contribution of alcohol use disorders to the burden of dementia in France 2008-13: A nationwide retrospective cohort study. Lancet Public Health.

[125] Sharma A., Brenner M., Jacob A., Marambaud P., Wang P. (2021). Extracellular CIRP Activates the IL-6Rα/STAT3/Cdk5 Pathway in Neurons. Mol. Neurobiol..

[126] Rajayer S.R., Jacob A., Yang W.L., Zhou M., Chaung W., Wang P. (2013). Cold-inducible RNA-binding protein is an important mediator of alcohol-induced brain inflammation. PLoS ONE.

[127] Guerri C., Pascual M. (2010). Mechanisms involved in the neurotoxic, cognitive, and neurobehavioral effects of alcohol consumption during adolescence. Alcohol.

[128] Jacobus J., Tapert S.F. (2013). Neurotoxic effects of alcohol in adolescence. Annu. Rev. Clin. Psychol..

[129] Zuo W., Zhao J., Zhang J., Fang Z., Deng J., Fan Z., Guo Y., Han J., Hou W., Dong H. (2021). MD2 contributes to the pathogenesis of perioperative neurocognitive disorder via the regulation of α5GABA(A) receptors in aged mice. J. Neuroinflamm..

[130] Zhang Y., Liang X., Bao X., Xiao W., Chen G. (2022). Toll-like receptor 4 (TLR4) inhibitors: Current research and prospective. Eur. J. Med. Chem..

[131] Chen Y.D., Huang P.Y., Chiang C.S., Huang Y.S., Tang S.C. (2021). Generation and Role of Calpain-Cleaved 17-kDa Tau Fragment in Acute Ischemic Stroke. Mol. Neurobiol..

[132] Yan W., Wang C., Zhao Y., Jiang Y., Sun M. (2024). Involvement of Calpain in Neurovascular Unit Damage through Up-regulating PARP-NF-κB Signaling during Experimental Ischemic Stroke. Mol. Neurobiol..

[133] Liu Y., Yang G., Liu M., Zhang Y., Xu H., Mazhar M. (2025). Cinnamaldehyde and its combination with deferoxamine ameliorate inflammation, ferroptosis and hematoma expansion after intracerebral hemorrhage in mice. J. Neuroinflamm..

[134] Coll R.C., Robertson A.A., Chae J.J., Higgins S.C., Muñoz-Planillo R., Inserra M.C., Vetter I., Dungan L.S., Monks B.G., Stutz A. (2015). A small-molecule inhibitor of the NLRP3 inflammasome for the treatment of inflammatory diseases. Nat. Med..

[135] Montaner J., Mendioroz M., Delgado P., García-Berrocoso T., Giralt D., Merino C., Ribó M., Rosell A., Penalba A., Fernández-Cadenas I. (2012). Differentiating ischemic from hemorrhagic stroke using plasma biomarkers: The S100B/RAGE pathway. J. Proteom..

[136] Dragoș H.M., Stan A., Pintican R., Feier D., Lebovici A., Panaitescu P., Dina C., Strilciuc S., Muresanu D.F. (2023). MRI Radiomics and Predictive Models in Assessing Ischemic Stroke Outcome-A Systematic Review. Diagnostics.

[137] Bader E.R., Pana T.A., Barlas R.S., Metcalf A.K., Potter J.F., Myint P.K. (2022). Elevated inflammatory biomarkers and poor outcomes in intracerebral hemorrhage. J. Neurol..

[138] Masotti L., Ceccarelli E., Forconi S., Cappelli R. (2005). Prognostic role of C-reactive protein in very old patients with acute ischaemic stroke. J. Intern. Med..

[139] Arrich J., Schütz N., Oppenauer J., Vendt J., Holzer M., Havel C., Herkner H. (2023). Hypothermia for neuroprotection in adults after cardiac arrest. Cochrane Database Syst. Rev..

[140] Zhou K.Q., Davidson J.O., Bennet L., Gunn A.J. (2020). Combination treatments with therapeutic hypothermia for hypoxic-ischemic neuroprotection. Dev. Med. Child Neurol..

[141] Banks W.A. (2015). Peptides and the blood-brain barrier. Peptides.

[142] Liu W., Wu D.H., Wang T., Wang M., Xu Y., Ren Y., Lyu Y., Wu R. (2025). CIRP contributes to multiple organ damage in acute pancreatitis by increasing endothelial permeability. Commun. Biol..

[143] Liu W., Ren Y., Wang T., Wang M., Xu Y., Zhang J., Bi J., Wu Z., Zhang Y., Wu R. (2024). Blocking CIRP protects against acute pancreatitis by improving mitochondrial function and suppressing pyroptosis in acinar cells. Cell Death Discov..

[144] Xu Q., Wang M., Guo H., Liu H., Zhang G., Xu C., Chen H. (2021). Emodin Alleviates Severe Acute Pancreatitis-Associated Acute Lung Injury by Inhibiting the Cold-Inducible RNA-Binding Protein (CIRP)-Mediated Activation of the NLRP3/IL-1β/CXCL1 Signaling. Front. Pharmacol..

[145] Wu Z., Liu X., Huang W., Chen J., Li S., Chao J., Xie J., Liu L., Yang Y., Wu X. (2024). CIRP increases Foxp3(+) regulatory T cells and inhibits development of Th17 cells by enhancing TLR4-IL-2 signaling in the late phase of sepsis. Int. Immunopharmacol..

[146] Shimizu J., Murao A., Nofi C., Wang P., Aziz M. (2022). Extracellular CIRP Promotes GPX4-Mediated Ferroptosis in Sepsis. Front. Immunol..

[147] Zhang F., Brenner M., Yang W.L., Wang P. (2018). A cold-inducible RNA-binding protein (CIRP)-derived peptide attenuates inflammation and organ injury in septic mice. Sci. Rep..

[148] Li D., Jin S., Teng X., Wang P., He K., Cao L., Du J., Guo Q., Xiao L., Xue H. (2025). Hydrogen sulfide attenuates sepsis-induced cardiac dysfunction in infant rats by inhibiting the expression of cold-inducible RNA-binding protein. Biosci. Rep..

[149] Tang R.X., Xie X.J., Xiong Y., Li S., Luo C., Wang Y.G. (2024). C23 ameliorates carbon tetrachloride-induced liver fibrosis in mice. World J. Hepatol..

[150] Bolourani S., Sari E., Brenner M., Wang P. (2022). The role of eCIRP in bleomycin-induced pulmonary fibrosis in mice. PLoS ONE.

[151] Gao Y., Liu H., Zhou J., Guo M., Sun J., Duan M. (2023). The Protective Effect of C23 in A Rat Model of Cardiac Arrest And Resuscitation. Shock.

[152] Liu W., Fan Y., Ding H., Han D., Yan Y., Wu R., Lv Y., Zheng X. (2021). Normothermic machine perfusion attenuates hepatic ischaemia-reperfusion injury by inhibiting CIRP-mediated oxidative stress and mitochondrial fission. J. Cell. Mol. Med..

[153] Zhang F., Hu Z., Jacob A., Brenner M., Wang P. (2025). An eCIRP inhibitor attenuates fibrosis and ferroptosis in ischemia and reperfusion induced chronic kidney disease. Mol. Med..

[154] McGinn J., Zhang F., Aziz M., Yang W.L., Nicastro J., Coppa G.F., Wang P. (2018). The Protective Effect of A Short Peptide Derived From Cold-Inducible RNA-Binding Protein in Renal Ischemia-Reperfusion Injury. Shock.

[155] Zheng X., Fan Y., Li J., Ma T., Li Y., Wang Q., Yan Y., Liu W. (2022). Change in Oxidative Stress and Mitochondrial Dynamics in Response to Elevated Cold-Inducible RNA-Binding Protein in Cardiac Surgery-Associated Acute Kidney Injury. Oxidative Med. Cell. Longev..

[156] Zhang X., Wang S., Wang W., Song L., Feng S., Wang J., Kang T., Yang P., Wang N., Yang P. (2022). Extracellular CIRP Upregulates Proinflammatory Cytokine Expression via the NF-kappaB and ERK1/2 Signaling Pathways in Psoriatic Keratinocytes. Mediat. Inflamm..

[157] Wang T., Wang M., Liu W., Zhang L., Zhang J., Zhao J., Wu Z., Lyu Y., Wu R. (2025). Intracellular CIRP promotes liver regeneration via STAT3 signaling pathway activation after partial hepatectomy in mice. Int. J. Mol. Med..

[158] Xu X., Kwon J., Yan R., Apio C., Song S., Heo G., Yang Q., Timsina J., Liu M., Budde J. (2025). Sex Differences in Apolipoprotein E and Alzheimer Disease Pathology Across Ancestries. JAMA Netw. Open.

[159] Huang X., Li S., Qiu N., Ni A., Xiong T., Xue J., Yin K.J. (2024). Sex and Age-Dependent Effects of miR-15a/16-1 Antagomir on Ischemic Stroke Outcomes. Int. J. Mol. Sci..

